# Effects of Type of Agreement Violation and Utterance Position on the Auditory Processing of Subject-Verb Agreement: An ERP Study

**DOI:** 10.3389/fpsyg.2016.01276

**Published:** 2016-08-30

**Authors:** Sithembinkosi Dube, Carmen Kung, Varghese Peter, Jon Brock, Katherine Demuth

**Affiliations:** ^1^Department of Linguistics, Macquarie UniversitySydney, NSW, Australia; ^2^ARC Centre for Cognition and its Disorders, Macquarie UniversitySydney, NSW, Australia; ^3^MARCS Institute for Brain, Behaviour and Development, Western Sydney UniversityPenrith, NSW, Australia; ^4^Department of Cognitive Sciences, Macquarie UniversitySydney, NSW, Australia; ^5^Santa Fe InstituteSanta Fe, NM, USA

**Keywords:** subject-verb agreement, utterance position, type of agreement violation, ERPs, auditory modality

## Abstract

Previous ERP studies have often reported two ERP components—LAN and P600—in response to subject-verb (S-V) agreement violations (e.g., *the boys* ^*^runs). However, the latency, amplitude and scalp distribution of these components have been shown to vary depending on various experiment-related factors. One factor that has not received attention is the extent to which the relative perceptual salience related to either the *utterance position* (verbal inflection in utterance-medial vs. utterance-final contexts) or the *type of agreement violation* (errors of omission vs. errors of commission) may influence the auditory processing of S-V agreement. The lack of reports on these effects in ERP studies may be due to the fact that most studies have used the visual modality, which does not reveal acoustic information. To address this gap, we used ERPs to measure the brain activity of Australian English-speaking adults while they listened to sentences in which the S-V agreement differed by type of agreement violation and utterance position. We observed early negative and positive clusters (AN/P600 effects) for the overall grammaticality effect. Further analysis revealed that the mean amplitude and distribution of the P600 effect was only significant in contexts where the S-V agreement violation occurred utterance-finally, regardless of type of agreement violation. The mean amplitude and distribution of the negativity did not differ significantly across types of agreement violation and utterance position. These findings suggest that the increased perceptual salience of the violation in utterance final position (due to phrase-final lengthening) influenced how S-V agreement violations were processed during sentence comprehension. Implications for the functional interpretation of language-related ERPs and experimental design are discussed.

## Introduction

Most native-speaking adults are able to instantaneously recognize whether a sentence is grammatical or not during sentence comprehension. This is an amazing feat given that the processes underlying sentence comprehension are by no means simple (e.g., Nichols, 1986; Nicol et al., 1997; Pearlmutter et al., 1999; Rayner and Clifton, 2009; Wagers et al., 2009). For example, when presented with sentences such as “*The boy often cooks on the stove*” or “*The boys often cook on the stove*,” English speakers must keep track of the grammatical information (i.e., number) of the subject noun phrase in order to determine which verb form qualifies as a suitable continuation of the sentence. Thus, in the first sentence, the verb will take the 3rd person singular −s (3SG) inflection, whereas in the second sentence, the verb remains uninflected. Failure to use the appropriate verb form results in ungrammatical sentences, as in “*The boy often* ^*^cook on the stove” and “^*^The boys often ^*^cooks on the stove.” This phenomenon of establishing grammatical relations between the subject and the verb is known as subject-verb agreement (S-V agreement).

Knowledge of the S-V agreement rule is thus considered to facilitate successful sentence comprehension. However, recent studies suggest that there are a number of other factors, such as type of morphological feature or syntactic complexity of the morpheme, that interact with the processing of grammatical information during on-line sentence comprehension (for a review, see Molinaro et al., 2011). One factor that has received relatively little attention in agreement processing studies is relative perceptual salience due to (i) the prosodic context of the target word (utterance-medial vs. utterance-final) and (ii) the overtness of the violation (errors of omission vs. commission). The present study therefore examined how effects of perceptual salience due to *utterance position* and *type of agreement violation* may modulate the neural responses to S-V agreement violations during on-line speech comprehension. The findings contribute to our understanding of the types of information that influence on-line sentence comprehension, and have implications for study design.

While it is not yet known how perceptual salience may impact on the neural responses to S-V agreement, there is abundant evidence that the position of the target verb in the utterance modulates young children's production of grammatical morphemes (e.g., Song et al., 2009; Theodore et al., 2011, 2012). For example, Song et al. (2009) observed that children typically produce 3rd person singular morphemes more reliably when the verb occurs utterance finally compared to utterance medially. This is thought to be due to the fact that syllables (and morphemes) occurring utterance finally are longer in duration than those that occur utterance medially (Wightman et al., 1992; Hsieh et al., 1999; Christophe et al., 2003, 2004; Oller, 2005; Wagner and Watson, 2010). As a result, these longer utterance-final morphemes might also be perceived better than the utterance-medial ones.

To test this hypothesis, Sundara et al. (2011) investigated 2-year-olds' perceptual sensitivity to grammatical (inflected) vs. ungrammatical (uninflected) 3rd person singular verbs in utterance-final versus utterance-medial position in an auditory visual-fixation task (e.g., *Now he* *cries* vs.^*^Now he *cry*; *He* *cries* *now* vs.^*^He *cry* *now*). As expected, infants showed a difference in looking times to the grammatical vs. ungrammatical sentences when the verb and morpheme occurred utterance finally, but not utterance medially. They interpreted these findings to suggest that the increased duration of the −s morpheme at the end of the utterance provides extra acoustic cues for listeners, enhancing infants' ability to detect its presence, and ungrammatical absence. That is, infants were more sensitive to the missing morpheme utterance finally compared to utterance medially due to the greater perceptual salience of the morpheme in durationally longer utterance-final position. However, Sundara et al. (2011) did not explore whether children would be equally sensitive to grammatical violations involving errors of commission (*Now they* *cry* vs.^*^Now they cries; They *cry* *now* vs.^*^They *cries* *now*).

Given that both errors of omission and commission result in S-V agreement violations, we would expect listeners to be equally sensitive to the grammatical violation. However, there are a number of reasons to assume that listeners might be more sensitive to errors of commission compared to errors of omission. One of the assumptions is that listeners often perceive speech sounds that they expect to hear even when they are physically absent from the stimuli, that is, phoneme restoration (Warren, 1970). This may make omission errors more difficult to detect than commission errors in which an unexpected morpheme is inserted into the speech. Another related assumption is that with auditory presentation, the perception and identification of the morpheme may be dependent on its physical characteristics, which may in turn affect the detection of agreement violations. Thus, the mere presence of the superfluous −s morpheme in the errors of commission makes the violation more overt compared to errors of omission. Listeners might therefore be more sensitive to the overt error. However, to our knowledge, there is no empirical evidence showing how effects of auditory perceptual salience due to utterance position or type of agreement violation influence neural responses to S-V agreement processing during on-line speech comprehension.

One of the tools ideally suited for exploring the different kinds of information that modulate on-line sentence comprehension is the event-related potentials (ERPs). The ERPs are characteristic patterns of voltage change extracted from brain electrical activity recorded on the scalp by time-locking the electroencephalogram (EEG) to the presentation of the stimuli (Luck, 2014). The excellent temporal resolution of ERPs allows exploration of the nature and timing of the processes that underlie the on-line computation of grammatical agreement. Researchers can determine if the processes are qualitatively or quantitatively different by comparing the ERP waveforms in terms of their polarity, amplitude, latency, and scalp distribution. Evidence from ERP studies demonstrates that native-speaking adults are exquisitely sensitive to S-V agreement violations (Molinaro et al., 2011). There are two ERP components that have been widely associated with the processing of S-V agreement violation in native-speaking adults.

The first is a negativity that often occurs between 300 and 500 ms after the onset of the violation and has been observed to have a left anterior scalp distribution known as left anterior negativity (LAN) (e.g., Osterhout and Holcomb, 1992; Friederici et al., 1993; Coulson et al., 1998; Hahne and Friederici, 1999; Gunter et al., 2000; Kaan and Swaab, 2002). The LAN is understood to reflect the detection of morpho-syntactic violations (Friederici et al., 1993; Osterhout et al., 1994, 1996; Hagoort et al., 2003; Bornkessel and Schlesewsky, 2006; Kos et al., 2010; Batterink and Neville, 2013). The second ERP component is a positivity known as the P600, occurring between 500 and 1000 ms after violation onset and often observed with a centro-posterior scalp distribution (e.g., Osterhout and Mobley, 1995) or with a broad scalp distribution (e.g., Molinaro et al., 2011). The P600 has been observed after the LAN (a biphasic LAN/P600 effect) or on its own (e.g., Osterhout and Mobley, 1995; Coulson et al., 1998; Hagoort and Brown, 2000; Kaan et al., 2000) and is generally understood to reflect syntactic reanalysis or repair (e.g., Hahne and Friederici, 1999, 2002; Gunter et al., 2000).

Based on the evidence from previous studies that correlated morphosyntactic processing to the LAN and the P600, Friederici (2002) proposed a neuro-cognitive model of auditory sentence comprehension. This model is influenced by the syntax-first models of sentence comprehension which assume that syntactic information is processed autonomously, prior to any other (non-syntactic) information (e.g., Frazier and Fodor, 1978). The syntax-first models do not accommodate the view that syntactic and other types of information interact at each stage of language processing as assumed by the interactive processing models (e.g., Trueswell et al., 1993, 1994; Pickering and Garrod, 2007, 2013). However, Friederici (2002) argues that both autonomous processing and interactive processing, hold in principle, but describe different processing phases during language comprehension (i.e., early versus late). Thus, according to Friederici's model, the early stages of sentence comprehension entail syntactic categorisation, morphosyntactic segmentation and thematic role assignment; these processes are correlated to the ELAN, LAN, and N400 effects, respectively (see also, Friederici, 2011). On the other hand, the late stage of syntactic re-analysis entails the integration of other information relevant for the interpretation of the sentence; this process is correlated to the P600 effect. This model thus assigns a modular-specific functional interpretation to the LAN and P600 components. Furthermore, the proposition of this model suggests that the LAN would be a more reliable and stable component compared to the P600.

However, Friederici's model of sentence comprehension is not explicit on whether or how the nature of incoming syntactic and other types of information may modulate the LAN and P600 effects. As a result, the model has been challenged by studies which have observed these ERP effects to vary in their presence, latency, amplitude, and distribution as a function of the characteristics of the morphosyntactic elements in question. For example, some studies investigating agreement processing, in languages other than English, have reported an N400 effect instead of the typical LAN effect (e.g., Wicha et al., 2004). Others did not observe the LAN (e.g., Osterhout et al., 1994; Hagoort and Brown, 2000; Kaan et al., 2000; Kos et al., 2010). On the other hand, while the P600 effect is often reported for agreement violations, some studies have not reported it (e.g., O'Rourke and Van Petten, 2011). This variable realization of the LAN and P600 effects has resulted in some scholars questioning the modular functional interpretation of these ERP components (for discussion, see Kaan and Swaab, 2002; Bornkessel-Schlesewsky et al., 2015; Tanner, 2015). However, despite the ongoing debate about the functional significance of the LAN and P600 effects (see also, Kolk and Chwilla, 2007; Kuperberg, 2007), there is generally a strong correlation between grammatical violations and the presence of the LAN and/or P600 in native-speaking adults (Molinaro et al., 2011). In the following paragraphs, we take a closer look at previous ERP studies that have investigated S-V agreement processing involving inflectional violations, as summarized in Table 1.

**Table 1:** **A summary of previous ERP studies on inflectional S-V agreement violation processing**.

**Study (Language)**	**Modality**	**Type of agreement violation**	**Utterance Position**	**Example of stimuli**	**ERP Effect/latency (ms)**	
					**Negativity P600**	**Negativity P600**
Kutas and Hillyard, 1983 (English)	Visual	Omission	Medial	As a turtle grows its shell grows/^*^grow too.	LAN 300–600	Not reported
Osterhout and Mobley, 1995 (English)	Visual	Commission	Medial	The elected officials hope/^*^hopes.…	LAN 300–500	Centro-posterior 500–800
Osterhout et al., 1996 (English)	Visual	Commission	Medial	The doctors believe/^*^believes ….…	No negativity	Centro-posterior 500–800
Coulson et al., 1998 (English)	Visual	Omission and commission collapsed	Medial	Every Monday he mows/^*^mow the…. They sun/^*^suns themselves on ….…	LAN 300–500	Anterior-posterior 500–800
Kaan et al., 2000 (English)	Visual	Commission	Medial	Emily wonders whether the performers in the concert imitate/^*^imitates a.…	No negativity	Central maximum 500–700 Posterior maximum 700–900
Shen et al., 2013 (English)	Auditory	Omission	Medial	Larry pushes/^*^push his ……	AN 150–300	Posterior 700–900
Hagoort and Brown, 2000 (Dutch)	Visual and auditory	Substitution	Medial	The spoilt child throws/^*^throw ……(Het verwende kind gooit/^*^gooien …)	No negativity	Anterior-posterior 500–700 Posterior 700–900
Hasting and Kotz, 2008 (Germany)	Auditory	Substitution	Final	He bowls/^*^bowl*.* (Er kegelt/^*^kegelst) You bowl/^*^ bowls*.* (Du kegelst/^*^kegelt)	LAN 100–300	Centro-posterior 300–800
De Vincenzi et al., 2003 (Italian)	Visual	Omission and commission collapsed	Medial	The old waiter serves/^*^serve with …(Il cameriere anziano serve/^*^servono….) The skilled butchers cut/^*^cuts ……. (I macellai esperti tagliano/^*^taglia…)	LAN 340–400	Posterior 500–700
Kos et al., 2010 (Dutch)	Visual	Substitution	Medial	The spoiled child ^^*^^throw ……(Het verwende kind gooit/^*^gooien …)	No negativity	Centro-posterior 500–900

* *Ungrammatical verb-form*.

A consistent finding across all the 10 studies in Table 1 is that S-V agreement violations elicited P600 effects, albeit with varying latencies, amplitudes and scalp distribution. However, only half of these studies have also reported a left anterior negativity (LAN) or anterior negativity (AN) preceding the P600. The variability of the LAN effects is often explained in terms of morphological feature differences, while that of the P600 is explained in terms of whether the task was passive or active (e.g., Kolk and Chwilla, 2007) or whether the violation was syntactically simple or complex (e.g., Kutas and Hillyard, 1983; O'Rourke and Van Petten, 2011). However, the studies highlighted in Table 1 also show that the P600 effects reported in these studies also vary due to a number of experimental-related factors which include modality of presentation, position of the violation, and the type of agreement violation used. For example, studies which used the visual modality reported a LAN with an onset latency around 300 ms, and a P600 around 500 ms. In contrast, studies that used the auditory modality reported ERP effects with earlier onset latencies. For example, (Shen et al., 2013) reported the LAN with an onset around 140 ms while Hasting and Kotz (2008) reported the LAN with an onset around 100 ms and a P600 with an onset latency around 300 ms.

These gradient effects on the latency of the negativity are generally assumed to reflect the ease of detecting the violation whereas those of the P600 reflect the speed of the revision or reanalysis of the violation (Friederici, 1998). Thus the different latencies observed between the visual and auditory modalities have been interpreted to suggest that modality of presentation impacts on the processing of S-V agreement violations (e.g., Hasting and Kotz, 2008). However, Hasting and Kotz have further noted that the time-locking point used in S-V agreement studies also matters, suggesting that time-locking at the onset of the morpho-syntactic violation instead of word onset may contribute to latency differences.

Besides the latency differences occurring between different modes of presentation, the scalp distribution and the size of the P600 component reported in previous studies also differ as a function of syntactic complexity. For example, the difference between the longer P600 effects (500–900 ms) with a centro-posterior distribution reported by Kos et al. (2010) and the shorter P600 effects (500–700 ms) with a posterior distribution reported by De Vincenzi et al. (2003) are interpreted to be a function of type of violation complexity. The differences observed in the scalp distribution and sizes of the components are assumed to reflect the degree to which the brain is engaged in syntactic reanalysis (e.g., Osterhout et al., 2004). The degree of brain involvement during sentence processing has been shown to be influenced by the level of syntactic integration difficulty (e.g., Kaan et al., 2000) or complexity of the syntactic structure involved (e.g., Coulson et al., 1998; Nevins et al., 2007; O'Rourke and Van Petten, 2011). These findings show that ERPs are ideal for identifying factors that modulate the processing of S-V agreement violations during sentence comprehension. However, they also indicate that different methodological aspects of the experiment influence the realization and interpretation of the ERP components.

The question of whether different types of agreement violation and utterance position influence the processing of S-V agreement violations is therefore important, given that these factors have been variably used in previous studies. However, the variability of the LAN and P600 effects has never been considered in light of the *type of agreement violation* (errors of omission vs. errors of commission) and *utterance position* (medial vs. final). For example, Osterhout and Mobley (1995) looked at errors of commission, i.e., superfluous addition of the 3SG, (e.g., *the officials* hope/^*^hopes….) occurring sentence medially, in a visual modality paradigm. They reported a left-anterior negativity (LAN) with an onset around 300 ms followed by a centro-posterior P600 with an onset around 500 ms. Similar biphasic LAN/P600 effects were observed in other studies that used the visual paradigm and sentence-medial position, although they looked at both errors of omission and commission that were collapsed together in the analysis (e.g., Coulson et al., 1998). In contrast, Shen et al. (2013) looked at errors of omission, i.e., omission of the 3SG, (e.g., *Larry* pushes/^*^push *his* …) occurring utterance-medially in an auditory modality paradigm. They reported a bilateral anterior negativity (AN) with an onset around 150 ms followed by a posterior P600 with an onset around 700 ms.

Similarly, early LAN effects were observed in Hasting and Kotz (2008), who investigated agreement violation processing in German, using the auditory modality. However, the P600 effects observed in their study had an early onset latency around 300 ms. Importantly, Hasting and Kotz's study differed from Shen et al. (2013) in that it looked at S-V agreement violations involving substitution errors that occurred in utterance-final position. So while it seems that modality of presentation modulated the ERP latencies in these studies, these effects are confounded with effects of errors of omission vs. commission. Moreover, we do not know if utterance-final S-V agreement violations in English will result in similar effects reported in Hasting and Kotz (2008) given that none of the previous ERP studies have investigated utterance-position effects on the processing of S-V agreement violations during on-line auditory sentence comprehension.

The foregoing discussion has thus motivated the purpose of the present study in two ways. The first is that, to date, most ERP studies of S-V agreement have presented stimuli in the visual modality, with participants viewing sentences presented one word at a time. While this allows precise time-locking to the onset of individual words and is relatively straightforward to implement, it is clearly very different to the typical reading experience. Visual presentation also limits research to participants who are fluent readers. It is thus unsuitable for studies of grammatical development in young children and other special populations, such as second language learners. Moreover, insights gained from studies in the visual modality may not readily translate to auditory presentation.

The second, which is linked to the first, is that the few ERP studies of S-V agreement that have been carried out in the auditory modality have used a range of stimulus manipulations and different languages, and perhaps as a result, have produced inconsistent results. The first, conducted by Hasting and Kotz (2008), investigated substitution errors occurring sentence medially, in German. They noted an early LAN with an onset around 100 ms and an early but long-lasting positive component with an onset latency around 300 ms. Subsequently, Shen et al. (2013) looked at errors of omission (e.g., *Larry* *pushes*/^*^push *his* …) occurring sentence medially in English sentences. They reported an early bilateral anterior negativity (AN) with an onset around 150 ms followed by a posterior P600 with an onset around 700 ms. It is possible that the different results may be due to the different experimental designs used in these studies, e.g., stimuli manipulation and utterance position. However, no ERP study has systematically explored these factors in the same study, to establish whether or how they may impact the neural responses to S-V agreement during on-line speech comprehension.

The aim of the present study, therefore, was to use ERPs to systematically explore the effects of type of agreement violation and utterance position on listeners' neural responses to S-V agreement violation in English. To achieve this, we recorded listeners' ERP responses to grammatical and ungrammatical sentences in which the S-V agreement violations differed according to the utterance position (medial vs. final) in which they occurred. Furthermore, the type of agreement violation differed depending on whether the 3SG −s was omitted (errors of omission) or superfluously added (errors of commission) as shown in Table 2.

**Table 2:** **Experimental stimuli design with examples**.

**Utterance position**	**Type of agreement violation**	**Example**
Medial	Omission	The boy often cooks/^*^cook on the stove
	Commission	The boys often cook/^*^cooks on the stove
Final	Omission	The boy often cooks/^*^cook
	Commission	The boys often cook/^*^cooks

* *Ungrammatical verb forms are marked in asterisks*.

The manipulation of the S-V agreement violations by type of agreement violation and utterance balanced design, as described by Steinhauer and Drury (2012). However, given that our study used speech stimuli, we made an important decision on how we paired the grammatical and ungrammatical sentences for analysis. Instead of comparing grammatical and ungrammatical sentences that only target verb (also known as target verb manipulation), as shown in Table 2, we compared sentences that had the same target verb but differed in context (also known as context manipulation) as shown in Table 3.

**Table 3:** **Experimental comparisons used for analysis**.

**Verb-form**	**Grammaticality**	**Position**	
		**Medial**	**Final**
With −S	Grammatical	The boy often **cooks** on the stove	The boy often **cooks**
	Ungrammatical (Commission)	The boys often ^*^cooks on the stove	The boys often ^*^cooks
Without −S	Grammatical	The boys often **cook** on the stove	The boys often **cook**
	Ungrammatical (Omission)	The boy often ^*^cook on the stove	The boy often ^*^cook

* *Ungrammatical verb forms are marked in asterisks*.

Thus, ungrammatical verb-forms without an −S (errors of omission) were compared with grammatical verb-forms without −S whereas the ungrammatical verb-forms with a superfluous −S (errors of commission) were compared with grammatical verb-forms with −S. The context manipulation comparisons thus avoided possible confounding effects of the acoustic presence/absence of the −S sound.

Based on previous findings from ERP studies on agreement processing, we predicted that S-V agreement violations would elicit a biphasic LAN/P600 effect. However, if effects of perceptual salience modulated listeners' sensitivity to the violations, we expected that listeners might be more sensitive to ungrammatical verb-forms with −S (errors of commission) than to ungrammatical verb-forms without −S (errors of omission) due to greater perceptual salience of the overt violation. We also hypothesized that S-V agreement errors occurring utterance-finally would elicit larger LAN/P600 effects compared to errors that occurred utterance-medially.

## Methods

### Ethics statement

The Ethics committee for Human Research at Macquarie University approved the experimental methods used in this study. Written informed consent was obtained from all participants before the experiment began.

### Participants

Twenty monolingual Australian-English speaking adults (age range: 18–25 years; mean: 22; 11 female, 9 male) participated in this study. Participants were recruited from the university student population. All completed a questionnaire on their developmental and linguistic history before participating in the study, and all were right-handed, with no clinical history of hearing or learning disorders. They received either course credits for participation or $20 if they did not require the course credits. Eight additional participants were excluded from the final analysis due to excessive ERP artifacts (e.g., as a result of sweating, or too much movement).

### Stimuli

The auditory stimuli included 50 CVC target verbs that could be used intransitively in both sentence medial and final positions (e.g., *The boy often cooks on the stove* vs. *The boy often cooks*). This ensured that all verbs could be used in both utterance-medial and utterance-final conditions, respectively. The sub-categorisation status of the verbs was verified by five native speakers of English. Only those verbs with high-medium frequency were selected to ensure familiarity and to facilitate processing. The criteria for lexical frequency was that the verbs had between 1–3 counts on the SUBLEX Log_10_CD (Hofmann et al., 2007). In addition, only those verbs that ended with the voiceless coda stops /p/, /t/, /k/ were selected to make sure that the inflected−s morpheme was always realized in the same allophonic condition (e.g., as /s/). This facilitated subsequent splicing of the materials and ensured that all similar items had the same morpheme length (see below). As the stimuli were later paired with a picture to provide a visual context while listening to the sentence, the verbs also had to be highly imageable.

**Figure 1:**
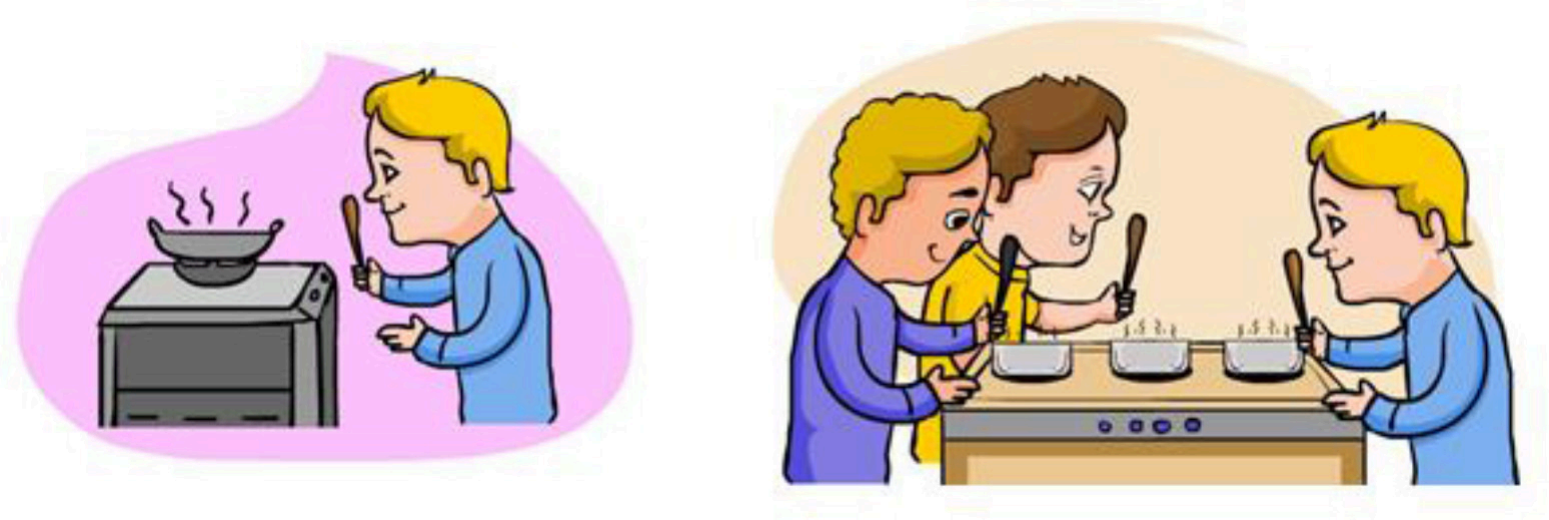
**Example of images used for the verb *cook/cooks***.

The verbs were inserted into carrier sentences that were composed of monosyllabic words, thereby controlling for utterance length and processing load. The carrier sentences had a singular vs. plural subject to enable manipulation of type of agreement violation (verb-form without −S/errors of omission vs. verb-form with −S/errors of commission). The verbs appeared in the middle vs. end of the carrier sentence to create the utterance-medial vs. utterance-final conditions, respectively (as shown above in Table 2). In the utterance-medial position, the verb was always followed by a preposition with a vowel onset to avoid masking of the morpheme in the preceding verb. All sentence stimuli were accompanied by cartoon pictures that were designed by a professional cartoonist (see example in Figure 1). The drawings had a constant level of visual complexity to avoid distracting details. The purpose of the pictures was to sustain participants' attention, and keep their eyes focused on the computer display to minimize head movement (muscle movements introduce artifacts to the ERP data).

This study employed a 2 × 2 × 2 design by crossing type of agreement and utterance position with grammaticality. Each verb therefore appeared in a total of eight conditions, resulting in 50 test items per condition and a total of 400 test items. In addition to the test items, there were 44 catch trials. All catch trials were grammatical and had the same structure as that of the target carrier sentences, but the verbs were not fully controlled for CVC structure (e.g., *eat*). These catch trials were used as a probe task in order to maintain participants' attention during the experiment (see Task and procedure for further details).

### Auditory stimulus preparation

All grammatical sentences were spoken by a female native speaker of Australian English who was trained in how to produce the sentences. To control for naturalness and intonational constancy, the sentences were read in response to a question and the accompanying picture. For example, all medial sentences were responses to a question like, “*What do the boys often do on the stove*? (Answer: *The boys often cook on the stove*). For the final conditions the question was “*What do the boys often do?* (Answer: *The boys often cook*). Medial and final conditions were separated into two lists and all sentences within the same list were recorded together. The sentences were recorded using Audacity (Audacity Team) in a sound-attenuated booth with a Behringer C2 microphone and a USBPre-2 amplifier. The recordings were digitized at a sampling rate of 44 KHz (16 bit; mono). Following the recording, the sentences were normalized using Audition C6 (Adobe Systems) and then extracted into individual sentences using Praat (Boersma and Weenink, 2012).

Instead of recording ungrammatical sentences, we created the stimuli by cross-splicing the grammatical productions from the onset of the verb, as shown in Table 4. All sound files were spliced at the zero-crossing from the beginning of the verb using Audition C6 (Adobe Systems). This procedure was meant to minimize the possibility of listeners using any early acoustic cues to distinguish between the grammatical and the ungrammatical condition. Previous studies using the auditory EEG paradigm have observed that recording ungrammatical structures, even with a trained speaker, introduces subtle but systematic slowing in production as well as intonation modifications (Hasting and Kotz, 2008; Royle et al., 2013). Therefore, the splicing procedure was used to avoid possible acoustic differences between grammatical and ungrammatical sentences before the point of violation. All stimuli were later rated for naturalness by a highly trained phonetician.

**Table 4:**
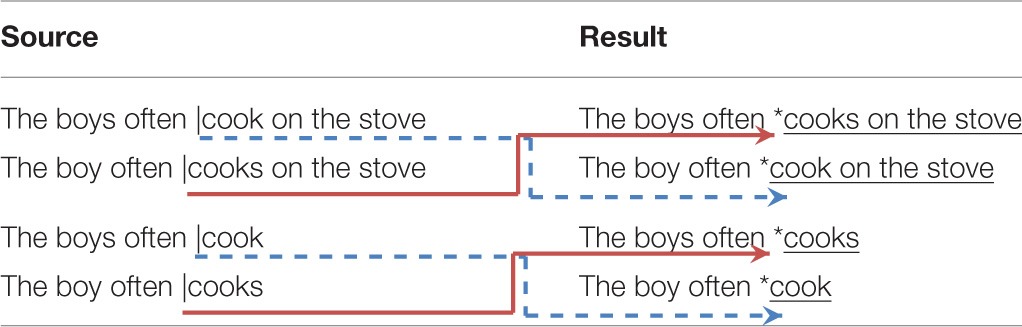
**Splicing points and procedure for creating ungrammatical stimuli**.

**Figure 2:**
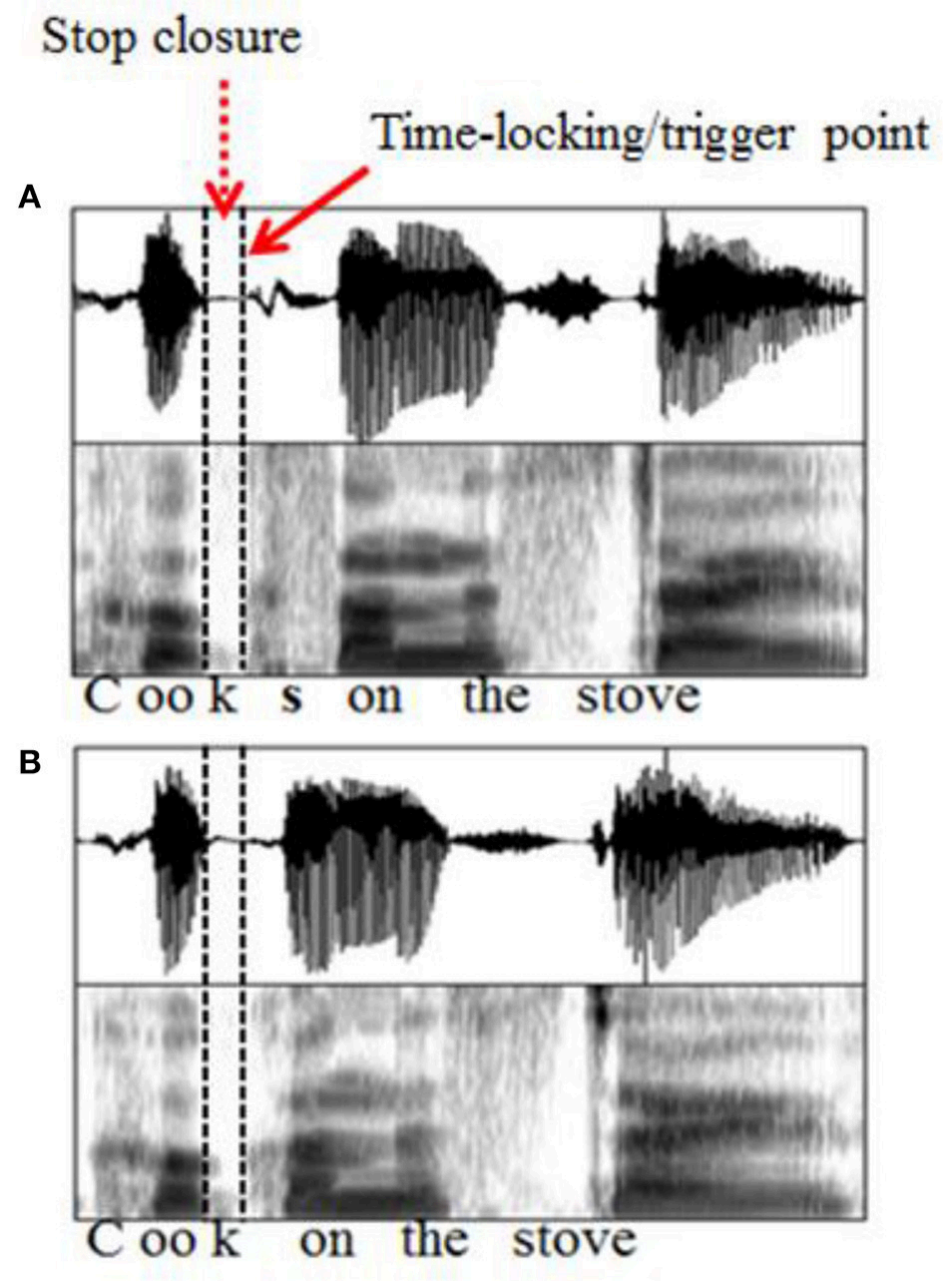
**Representative waveforms and spectrograms illustrating the time-locking point used for ERP analysis; (A) illustrates the inflected verb (cooks) and (B) the uninflected verb (cook)**. The dotted arrow indicates the stop closure of the oral-stop coda /k/ and the solid arrow indicates the end of stop closure that was used as the time-locking point in grammatical and ungrammatical experimental conditions.

After splicing the stimuli, we used Audition C6 (Adobe Systems) to examine the waveforms and insert triggers into the individual sound files. We systematically used the end of closure for the coda stops, instead of the end of burst release, as the time-locking point for all four conditions. This is because the burst release of some coda stops such as /t/ is not always clearly identifiable when followed by frication (i.e., the /s/ 3SG morpheme). By time-locking to the end of closure, we made sure that the time-locking points for grammatical and ungrammatical sentences were identical in all conditions. The spectrograms in Figure 2 illustrate the time-locking points for grammatical and ungrammatical conditions that had inflected and uninflected verb-forms. Having the same time-locking point ensured that the grammatical and ungrammatical conditions were comparable in terms of where and when the ERP violation effects appeared in both medial and final contexts.

Recall that one of the aims of this study was to explore the effects of perceptual salience on the sensitivity to S-V agreement violations. Critical to this effect is the prediction that 3SG −s will be longer utterance finally due to phrase-final lengthening. To ensure that this was the case we used Praat to conduct acoustic measures of frication duration across all 50 tokens of 3SG −s. As expected, the −s in utterance-final position was twice as long as the morpheme utterance medially, with a mean duration of 238 ms (SD 28 ms) compared to 114 ms (SD 22 ms). Paired *t*-tests were used to compare the duration of the −s in medial and final position, and as expected, this difference was statistically significant, *t*_(49)_ = −5.989, *p* < 0.001. This confirmed that the 3SG morpheme in utterance-final position was longer than that in utterance-medial condition.

### Task and procedure

Participants were fitted with an electrode cap (Easycap, Brainworks, GmbH) while seated in a comfortable plush chair at a distance of one meter from a CRT computer screen, in a dimly lit sound-attenuated and electromagnetically shielded room. EEG signals were recorded continuously as participants listened to sentences. They were instructed to listen attentively to all sentences and to immediately press a given response button when they heard the words “*cut/cuts”* or “*eat/eats”* in the sentence. These verbs were used as catch trials while the button-press task prevented participants from performing explicit grammaticality judgments. This probe task was therefore used to distract participants from concentrating on the grammaticality of the sentences without hindering the natural comprehension process (Dragoy et al., 2012).

The sentences and their matching pictures were presented using Presentation (Neurobehavioral Systems) which also recorded responses (hits, misses and false alarms) for the probe task. These behavioral responses helped us to determine if participants were attending to the task. The sentences were presented via two audio speakers, at an intensity of 75 dB SPL, while the matching images appeared on the screen. The speakers were positioned on the left and right of the computer screen.

The sentences were grouped into medial and final lists in which each list had two 10-min blocks. Each block had 111 sentences with accompanying pictures. The lists were presented separately to avoid mixing the medial and final conditions as they were of different word lengths. By blocking the presentation we also controlled for the possibility that the transitivity of the medial condition (verb + prepositional phrase) would influence participants' interpretation of final sentences, as they might then have expected a prepositional phrase in this condition as well. This was particularly important given that one of the aims of this study was to explore utterance position effects, we had to minimize any possible confounds. To control for presentation list effects, the order of the blocks was counterbalanced among the participants so that half of the participants heard the medial-final order first, and the other half had the final-medial order first.

Within each block, the order of sentence/picture presentation was pseudo-randomized with the constraint that the same verb did not occur consecutively. Two catch trials were presented at the beginning of the first block of each list and the presentation was pseudo-randomized with the constraint that they occur after five to eight consecutive target items within the block. A picture of an eye appeared on the screen ~1000 ms after the end of each sentence to control for eye blinks and remained on the screen for 1000 ms. Participants were asked to avoid blinking during the presentation of the sentences but to blink when the picture of an eye appeared on the screen. They were also asked to sit still during the presentation of the sentences to avoid movement artifacts during the EEG recording. The sentences had an inter-stimulus-interval of 3 s. A short break was taken at the end of each block. The duration of the break was determined by the participant. Altogether, the experiment lasted about 60 min.

### EEG data recording

The continuous EEG was recorded using SCAN 4.5 (Compumedics Ltd., USA) from 64 Ag/AgCl scalp electrodes mounted onto an electrode cap (Easycap, Brainworks, GmbH) in line with the International 10–20 system (Jasper, 1958: Fpz, Fz, FCz, Cz, CPz, Pz, POz, Oz, Fp1/2, F7/5/3/1/2/4/6/8, FT7/8, FC5/3/1/2/4/6, T7/8, C5/3/1/2/4/6, M1/2, TP7/8, CB1/2, CP5/3/1/2/4/6, P7/5/3/1/2/4/6/8, PO7/5/3/4/6/8, O1/2). Additional electrodes were placed above and below the left orbit and on the outer canthus of each eye to monitor electro-oculographic (EOG) activity with a bipolar recording. The ground electrode was positioned between Fpz and Fz. Electrode impedances were adjusted until they were below 10 kΩ. Electrical activity was recorded from both mastoids with the left mastoid (M1) serving as the online reference. The signal from the EEG was digitized at a sampling rate of 1000 Hz and filtered with a 0.05–100 Hz bandpass filter using a Neuroscan SynAmps2 DC Amplifier (Compumedics Ltd., USA).

### EEG data processing

The digitized data were processed off-line in Matlab (Version R2013b: MathWorks, Machussets, USA) using the Fieldtrip toolbox (Oostenveld et al., 2010: Version 2014-08-24). The data were epoched into trials of 1000 ms including a 100 ms pre-stimulus interval and then filtered with a Butterworth bandpass of 0.05–20 Hz for Independent Component Analysis (ICA) analysis. Extreme trials with amplitudes larger than ± 300 μV were removed before entering all trials into the ICA. The purpose of the ICA was to identify any components resembling eye blinks, horizontal eye movements, noisy channels and other focal artifacts. The identified components were then mathematically removed from the data and signals were back projected to the original unfiltered data. After ICA, each channel was re-referenced to the mean mastoids and baseline corrected using the 100 ms pre-stimulus interval. Trials with artifacts that exceeded 100 μV, with trends greater than 75 μV, or with abnormal distributions or improbable data exceeding five *SD*s, were also rejected. This procedure removed a total of 172 trials or (0.46% of all trials) from the eight experimental conditions: 21 medial-singular grammatical, 24 medial-singular ungrammatical (omission), 23 final-singular grammatical, 19 final-singular ungrammatical (omission), 21 medial-plural grammatical, 22 medial-plural ungrammatical (commission), 24 final-plural grammatical, and 18 final-plural ungrammatical (commission). There was no reliable difference between the numbers of rejected trials across conditions. The remaining trials in each of these conditions were averaged for each participant and grand averages were then computed for each of the conditions.

### EEG data analysis

An important decision in conducting data analysis was how to pair ungrammatical sentences with corresponding grammatical sentences. For example, the ungrammatical sentence “*The boys often cooks on the stove”* could be paired with “*The boys often cook on the stove,”* keeping the context consistent but changing the inflection on the verb. However, in auditory studies, this entails that grammaticality effects are confounded with differences in the acoustic content following the verb stem, in terms of both the presence/absence of the −s and the timing of the subsequent word. This in turn means that “grammaticality” effects on ERPs may arise even when participants are insensitive to the grammatical violation. We therefore chose instead to manipulate the context whilst keeping the verb inflection constant by comparing the grammatical vs. ungrammatical verbs across the singular and plural conditions. (e.g., *The boy often* *cooks* *on the stove vs. The boys often* *cooks* *on the stove*). This removes any potential acoustic confound following the verb. Although the context manipulation could itself present as an acoustic confound affecting the pre-stimulus baseline, this should be minimized by the intervening adverb (see Steinhauer and Drury, 2012) for discussion on effects of context/target manipulation on syntactic violation processing).

Another important decision was to objectively select an appropriate time-window for our auditory ERP data so that we could make direct comparisons across conditions. As discussed in the Introduction, different ERP latencies have been reported for studies using the visual and auditory modality (see literature review Table 1). Thus, instead of relying on a priori time windows associated with (L)AN or P600, we used non-parametric cluster-based permutation tests (Maris and Oostenveld, 2007) to identify time windows where significant effects of grammaticality were present in the grand averaged data collapsed across type (omission vs. commission) and position (utterance-medial vs. utterance-final). As described by Maris and Oostenveld (2007), the cluster-based permutation test first identifies sampling points with t-statistic exceeding a critical threshold (*p* < 0.05, two-tailed). Clusters are then formed by connecting significant sampling points on the basis of spatial and temporal adjacency. This is done separately for sampling points with positive and negative *t*-values. The maximum cluster-level test statistics (the sum of all individual *t*-values within a cluster) are then computed to generate permutation distributions, one for positive clusters and one for negative clusters, based on 1000 random partitions. The significance of a cluster is determined by whether it fell in the highest or the lowest 2.5th percentile of the corresponding distribution. To foreshadow our results, we identified two significant clusters, corresponding to the AN and P600.

**Figure 3:**
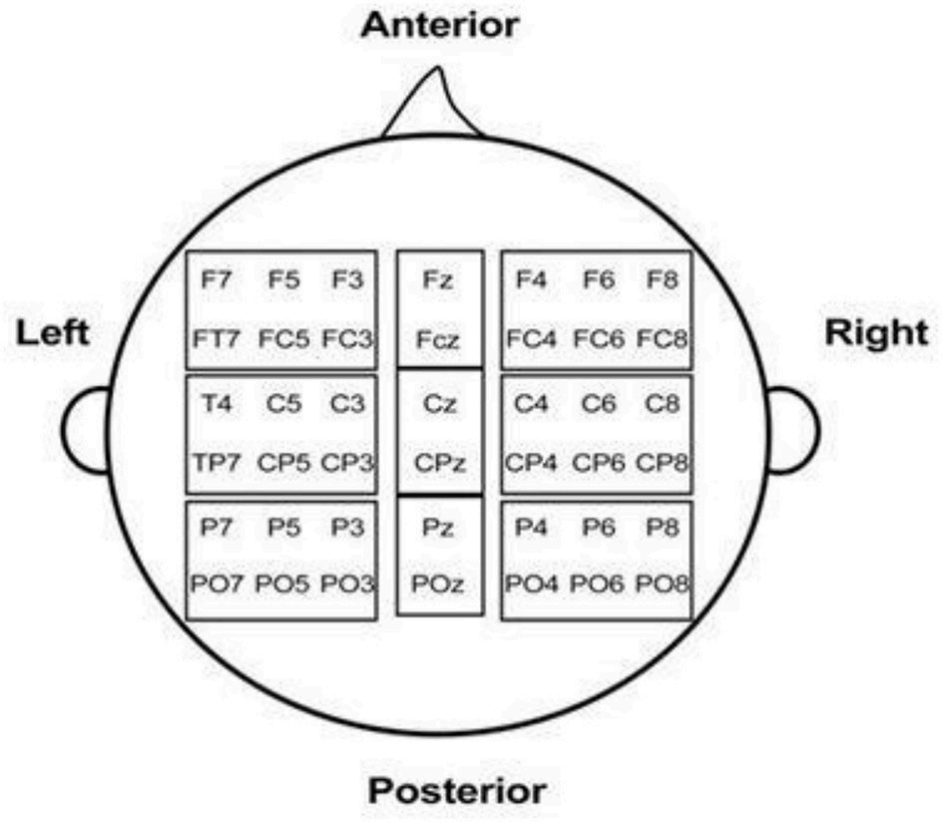
**Approximate placement for the electrodes included in the regions of interests (ROI) analysis for MANOVA**. The rectangles indicate the levels used to demacate the nine ROI [anterior midline (Fz, FCz), central midline (Cz, CPz), posterior midline (Pz, POz), anterior left (F7, F5, F3, FT7, FC5, FC3), central left (C3, C5, T7, CP3, CP5, TP7), posterior left (P7, P5, P3, PO7, PO5, PO3), anterior right (F4, F6, F8, FC4, FC6, FT8), central right (C4, C6, T8, CP4, CP6, TP8), posterior right (P8, P4, P6, PO4, PO6, PO8)].

For each component, we then performed a repeated-measures MANOVA using Grammaticality, Verb-form, Position, and Region of interest (ROI) as within-Subject variables. We defined nine ROIs, taking the means of electrodes in the parenthesis: [anterior midline (Fz, FCz), central midline (Cz, CPz), posterior midline (Pz, POz), anterior left (F7, F5, F3, FT7, FC5, FC3), central left (C3, C5, T7, CP3, CP5, TP7), posterior left (P7, P5, P3, PO7, PO5, PO3), anterior right (F4, F6, F8, FC4, FC6, FT8), central right (C4, C6, T8, CP4, CP6, TP8), posterior right (P8, P4, P6, PO4, PO6, PO8)]. These electrode groupings are illustrated in Figure 3.

We present the results from the cluster-based permutations first, and then the procedure and results for the MANOVAs. Note that the statistical analyses were performed on original unfiltered data, but for presentation purpose, the ERP waveforms presented in this paper were filtered using a 40-Hz low-pass filter.

## Results

### Effects of grammaticality

**Figure 4:**
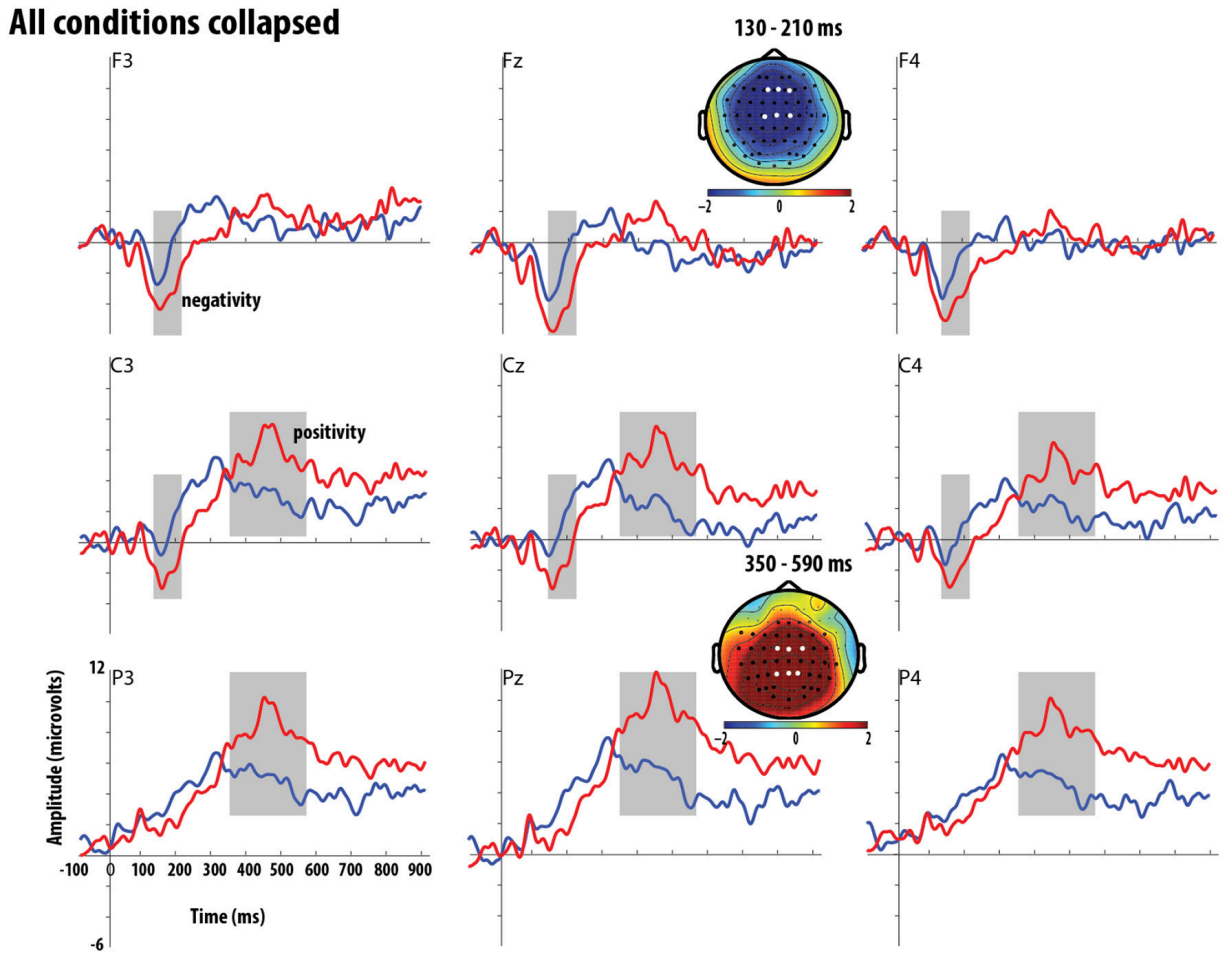
**Grand average ERP waveforms for grammatical and ungrammatical conditions across positions and type of agreement violation at the F3, Fz, F4, C3, Cz, C4, P3, Pz, and P4 electrodes and the topographic maps of the significant ERP effects**. The first row of the figure shows the anterior electrodes while the second row shows central electrodes and the third row shows the posterior electrodes. The ERPs are time-locked to the offset of the verb-stem (end of stop closure) and positivity is plotted upwards. The topographic maps show brain voltage distributions for the negative and positive clusters. These maps were obtained by interpolation from 64 electrodes and were computed by subtracting the grand averages of grammatical from the ungrammatical conditions. Electrodes in the significant clusters are highlighted with a black circle and the F3, Fz, F4, C3, Cz, C4, P3, Pz, and P4 electrodes in the significant clusters are highlighted with a white circle. Time-windows for significant clusters is highlighted in gray over the waveforms.

The primary goal of this study was to test if adult native English speakers would be sensitive to S-V agreement violations, as often reported in previous studies where there is generally a strong correlation between grammatical violations and the presence of the (L)AN and/or P600 in L1 adults (Molinaro et al., 2011). However, we further sought to explore if these responses would be modulated by the relative perceptual salience of S-V agreement violations as a function of *utterance position* (medial vs. final) and *type of agreement violation* where the verb-form occurred without an −S (errors of omission) or with a superfluous −S (errors of commission). We begin by reporting the results of the cluster-based permutation tests, which contrasted the grand average ERP waveforms of the grammatical condition with those of ungrammatical condition (collapsed over type of agreement and utterance position). The grammaticality effects are shown at nine representative electrodes (corresponding to locations F3, Fz, F4; C3, Cz, C4, and P3, Pz, P4 in a standard 10–20 set-up) in Figure 4, which also shows the topographic maps highlighting the distribution and time course of the significant clusters.

Visual inspection of the waveforms indicates that, relative to the grammatical verbs, ungrammatical verbs elicited a bilateral negative-going waveform over the anterior-central electrodes followed by a positive-going waveform over the central-posterior electrodes. Statistical analysis using cluster-based permutation tests revealed that contrasts observed for grammatical vs. ungrammatical verbs yielded a significant *negative* cluster (*p* = 0.036) between 130 and 210 ms in the anterior-central electrodes and a significant *positive* cluster (*p* < 0.0001) between 350 and 590 ms with a centro-posterior distribution.

### MANOVA: effects of type of agreement and utterance position

Waveforms for each of the four conditions are shown in **Figures 6–9**. Having established the presence of a Grammaticality effect, we then performed MANOVA on the two significant time windows (130–210 ms and 350–590 ms) to test the interaction between Grammaticality and type of agreement violation, utterance position, and ROI. The results of the two MANOVAs are reported in Table 5.

**Table 5:** **Omnibus MANOVA results across the 130–210 ms, and 350–590 ms time windows**.

	**210-390 ms**		**350-590 ms**	
**Effects**	**Pillai's trace**	***F*-value**	**Pillai's trace**	***F*-value**
Verb-form (1,19)	0.202	4.802^*^	0.250	6.325^**^
Pos. (1,19)	−	−	0.236	5.860^*^
Gram (1,19)	0.459	16.117^***^	0.380	11.642^***^
Verb-form. ^*^Pos (1,19)	−	−	0.342	9.875^***^
Verb-form. ^*^Gram (1,19)	−	−	−	−
Pos. ^*^Gram (1,19)	−	−	−	−
Verb-form.^*^Pos.^*^Gram (1,19)	−	−	−	−
Verb-form.^*^ROI (8,152)	0.710	3.672^*^	−	−
Pos. ^*^ROI (8,152)	0.686	3.280^*^	−	−
Verb-form.^*^Pos.^*^ROI (8,152)	−	−	−	−
Gram. ^*^ROI (8,152)	−	−	−	−
Verb-form. ^*^Gram.^*^ ROI (8,152)				
Pos.^*^Gram.^*^ ROI. (8,152)	−	−	0.705	3.593^*^
Verb-form.^*^Pos.^*^Gram.^*^ROI (8,152)	−	−	−	−

*Degrees of freedom are reported in parentheses. Pos. = Position, Gram. = Grammaticality, ROI = Regions of interest*.

*** p < 0.001;

** p < 0.05;

* *p = 0.05*.

### 130–210 ms time window

Consistent with the cluster analysis, the MANOVA showed a main effect of Grammaticality. However, the absence of any interactions involving Grammaticality indicates that the response to grammatical versus ungrammatical conditions was similar regardless of verb-form or positions.

The main effect of Verb-form and the interaction between Verb-form and ROI suggests that the response in this early time window differed significantly depending on the Verb-form (presence or absence of −S) independent of Grammaticality. Follow up pairwise *t*-tests revealed that, the *verb-forms without* −*S* elicited greater negativity compared to *verb-forms with* −*S* at the anterior-left region [*t*_(19)_ = −2.118, *p* < 0.05], and central-mid region [*t*_(19)_ = −3.818, *p* < 0.005].

The significant interaction between Position and ROI suggests that the mean amplitude of the negativity also differed across the electrodes depending on utterance position. Follow-up pairwise *t*-tests for the Position and ROI interaction revealed that verbs in utterance-medial position elicited more negativity compared to those in the utterance-final position at the front-mid region [*t*_(19)_ = −2.494, *p* < 0.05], anterior-left region [*t*_(19)_ = −2.438, *p* < 0.005), and anterior-right region [*t*_(19)_ = −3.017, *p* < 0.005].

These results thus suggest that the distribution of the negativity observed in the cluster-based permutation varied due to verb-form and utterance position. However, the absence of grammaticality interactions in this time window suggests that, although the mean amplitude of the negativity varied across the electrodes due to type of verb-form and utterance position, the difference between grammatical and ungrammatical conditions was the same in both types of verb-form and positions.

### 350–590 ms time window

**Figure 5:**
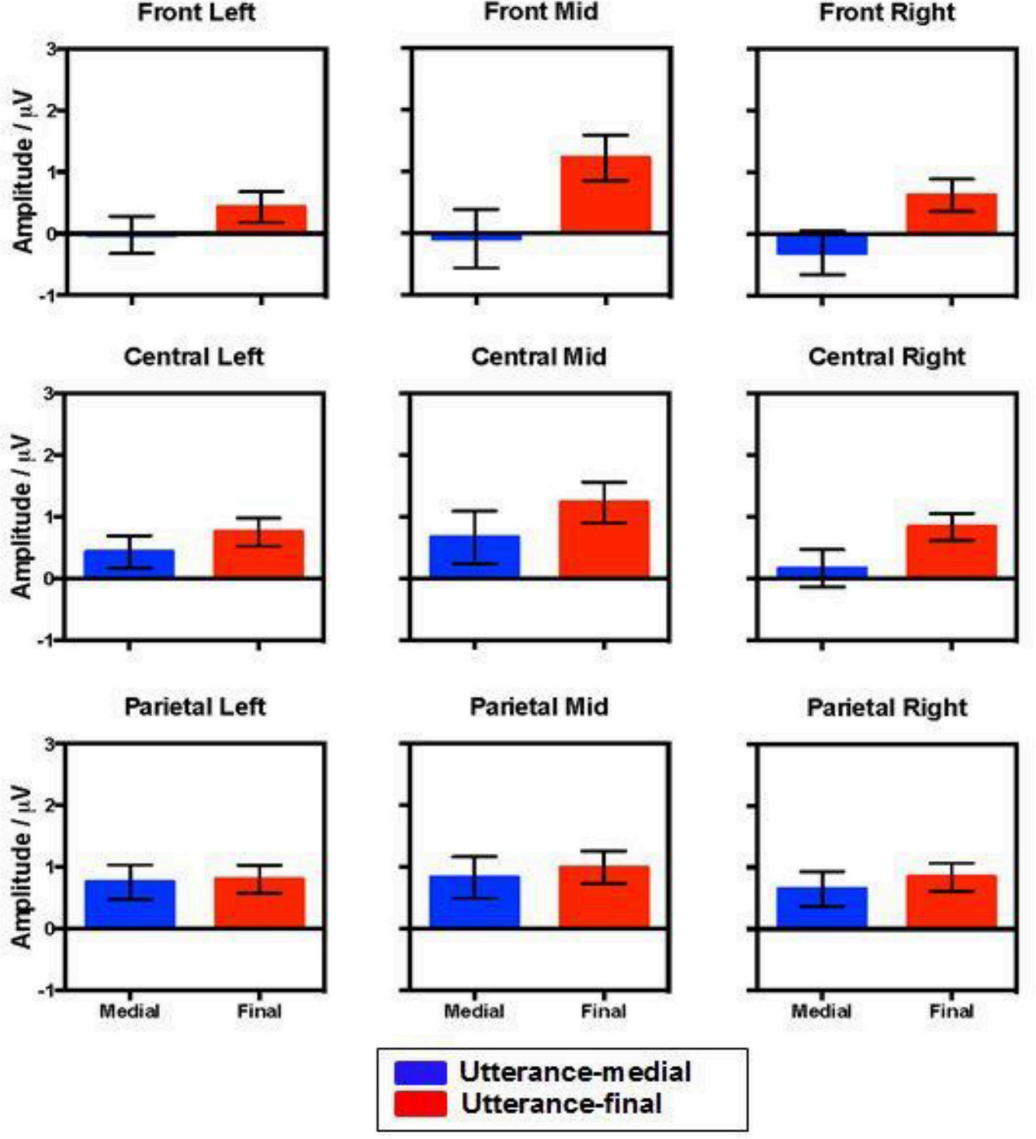
**Difference in mean amplitude between grammatical and ungrammatical conditions in the utterance-medial and utterance-final position across the 9 ROIs, showing error bars representing +1/−1 standard error**.

**Figure 6:**
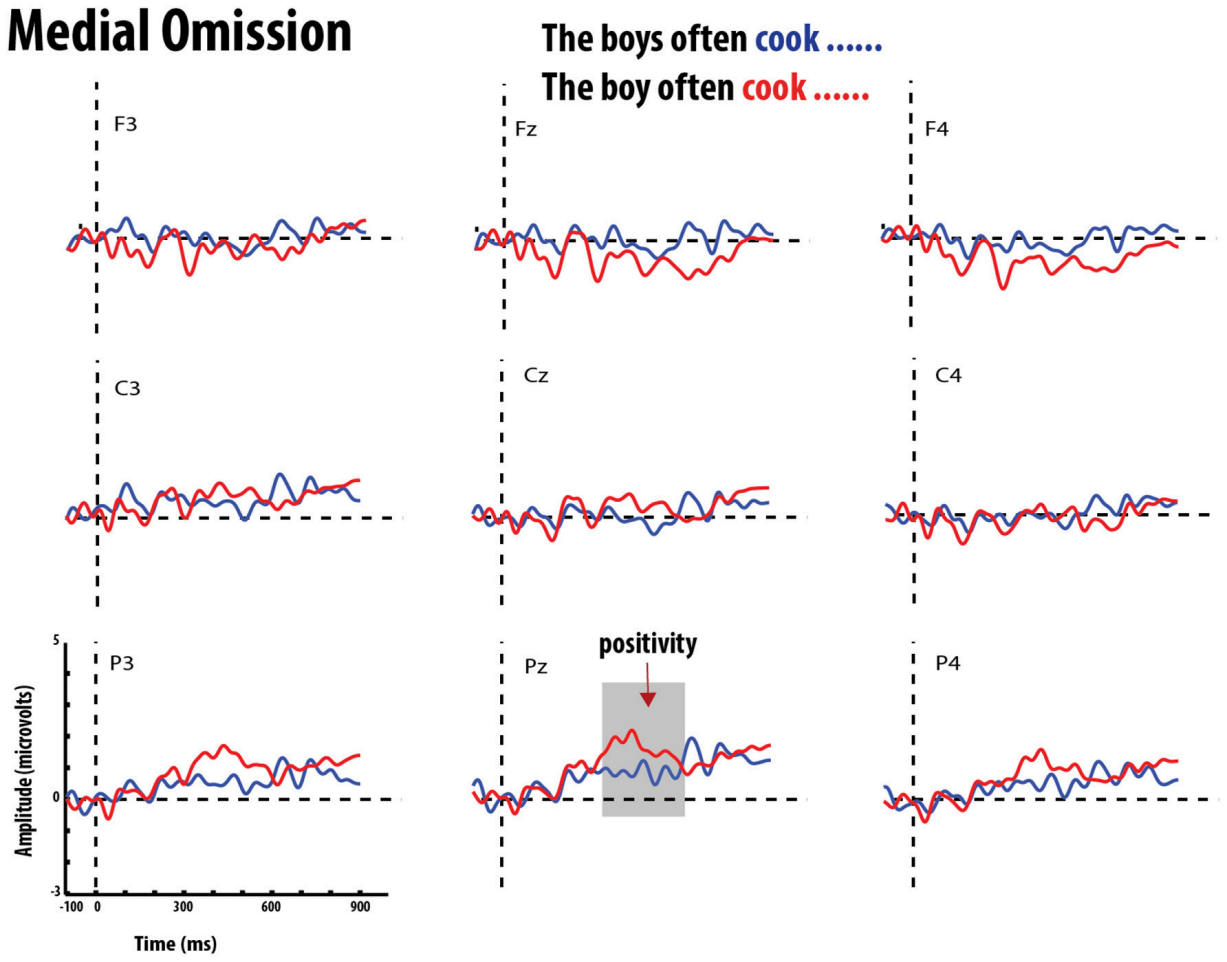
**Grand average event-related potentials elicited by errors of omission (red) and correct verb (blue) in medial position**. Gray bar highlights the significant time-window for the P600 effect.

**Figure 7:**
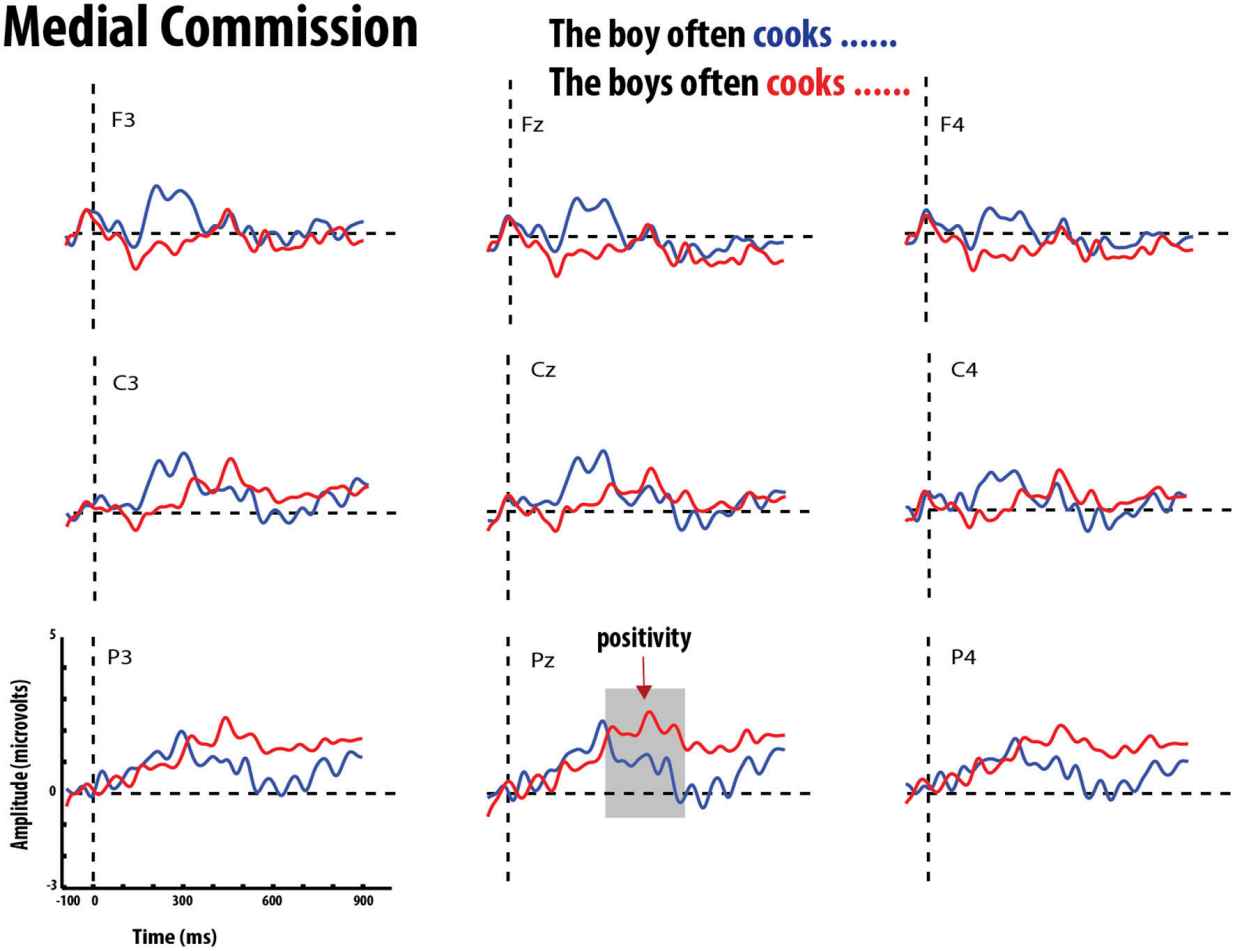
**Grand average event-related potentials elicited by errors of commission (red) and correct verb (blue) in medial position**. Gray bar highlights the significant time-window for the P600 effect.

**Figure 8:**
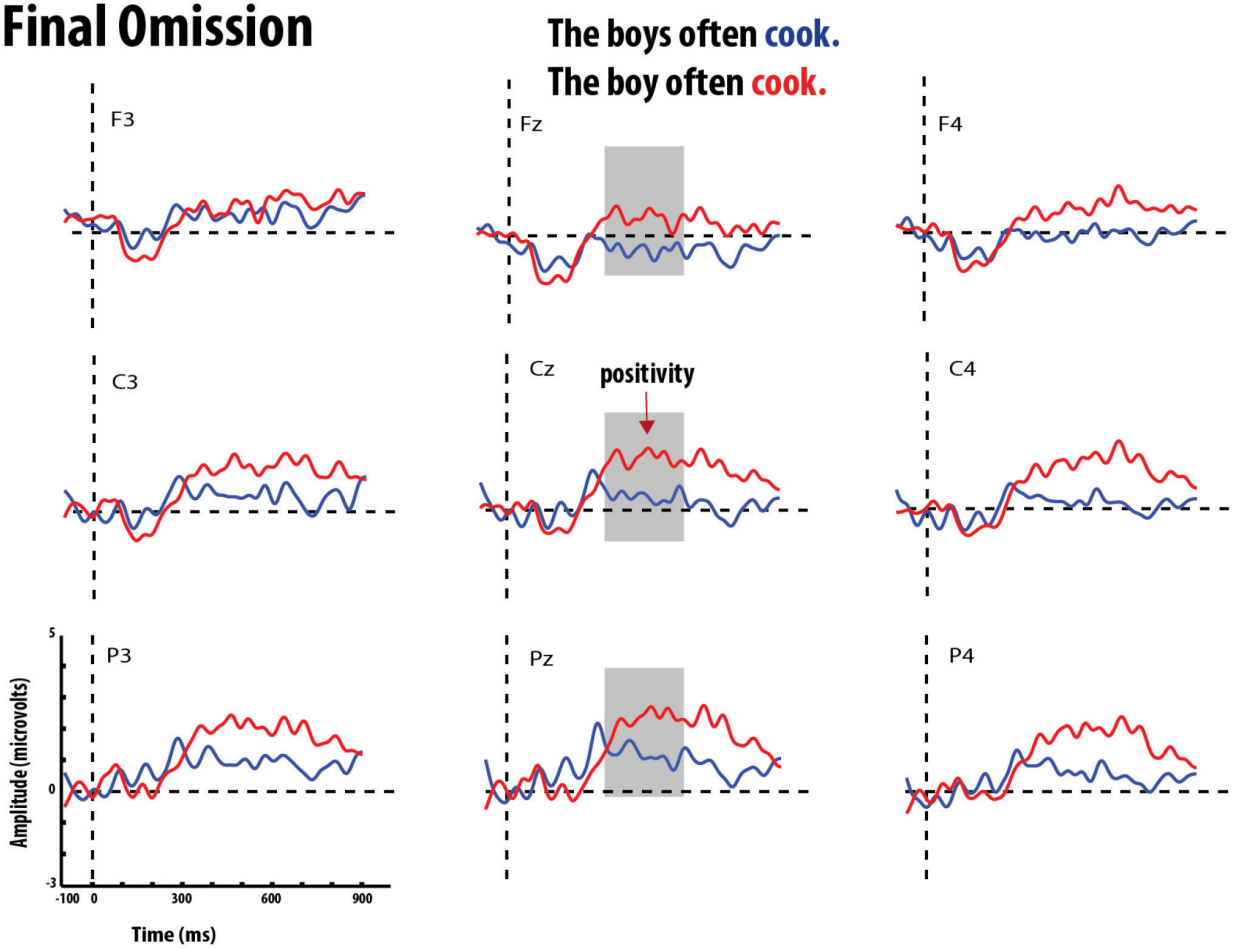
**Grand average event-related potentials elicited by errors of omission (red) and correct verb (blue) in final position**. Gray bar highlights the significant time-window for the P600 effect.

**Figure 9:**
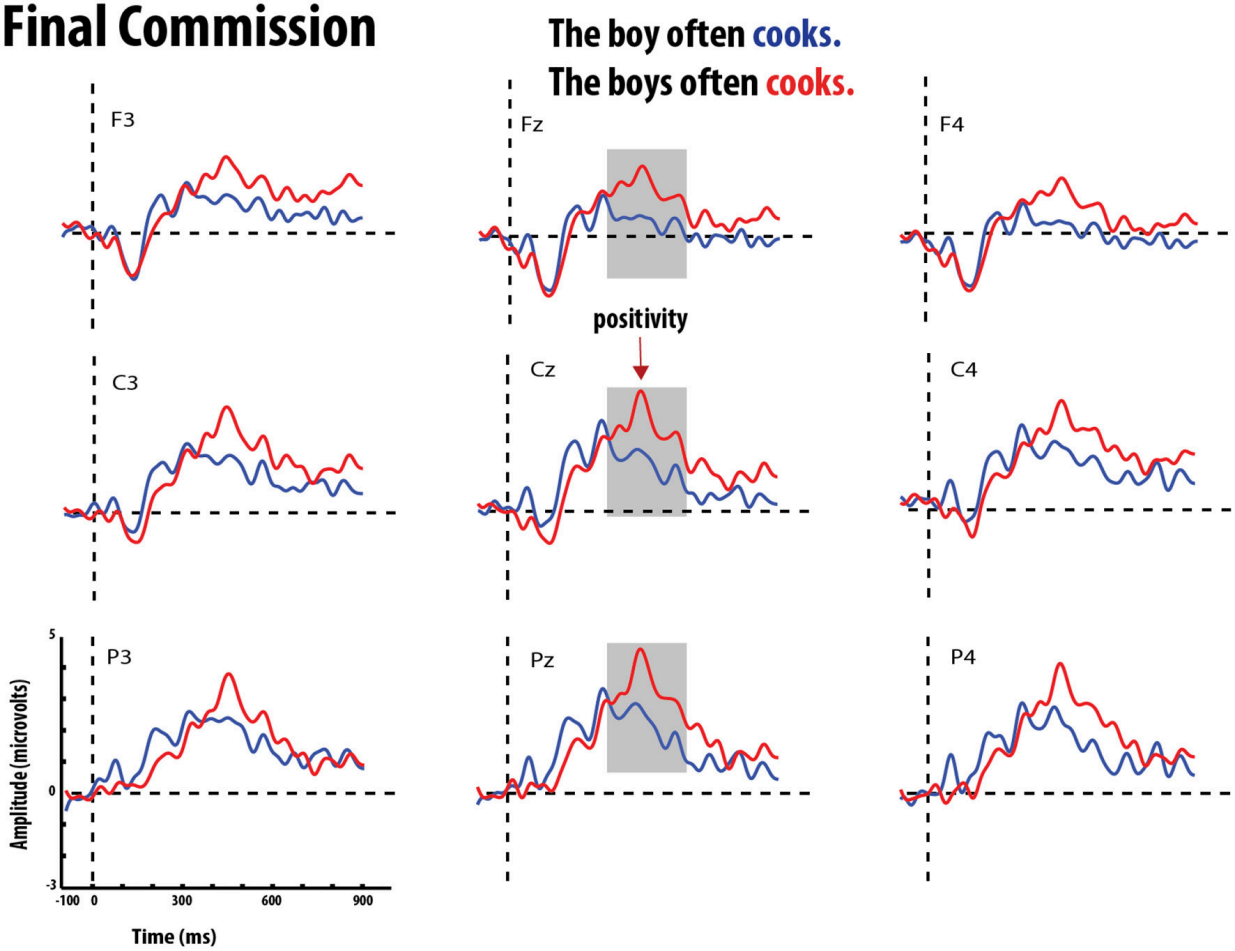
**Grand average event-related potentials elicited by errors of omission (red) and correct verb (blue) in final position**. Gray bar highlights the significant time-window for the P600 effect.

The statistical analysis for this time window showed main effects of Grammaticality, Verb-form and Position. There were no interactions between Grammaticality and Verb-form or Position. There was, however, a three-way interaction between Grammaticality, Position, and ROI. To test this, follow-up MANOVAS were performed on each ROI with Position and Grammaticality as within-subject factors. Results indicated that the interaction was significant in the anterior-mid region [Pillai's trace = 0.197, *F*_(1, 19)_ = 4.660, *p* < 0.05]. Further pairwise comparisons showed that the mean amplitude of the positivity for ungrammatical conditions was significantly greater in the utterance-final position (*M* = 1.685 μV, *SE* = 0.447) than in the utterance-medial position (*M* = 0.547 μV, *SE* = *0.294*), *t*_(19)_ = 3.152, *p* < 0.005. Figure 5 shows the mean amplitude of the Grammaticality effect in utterance-medial and utterance-final positions in the nine ROIs. This indicates that while the grammaticality effect had a broad distribution in the final position it was confined to central and parietal electrodes for the medial condition. This pattern is also reflected in the grand-averaged ERP waveforms for grammatical and ungrammatical conditions (errors of omission vs. commission) in the utterance-medial and utterance-final position in Figures 6–9.

The MANOVA also revealed an interaction between Verb-form and Position. This interaction suggests that the effect of Position differed depending on the Verb-form. Follow up pairwise *t*-tests revealed that the mean amplitude of the positivity elicited by verb-forms *with* −*S* was significantly greater in utterance-final position (*M* = 1.636 μV, *SE* = 0.228) than verb-forms in utterance-medial position (*M* = 0.318 μV, *SE* = 0.244), *t*_(19)_ = 3.152, *p* < 0.005. This suggests that participants were more sensitive to the verb-forms *with* −*S*, occurring in utterance-final position, regardless of grammaticality.

Overall, the interactions observed in this later time window indicate that the amplitude and distribution of the positivity was influenced by perceptual salience due to utterance-final lengthening.

## Discussion

This study used ERPs to investigate how Australian-English speaking adults processed S-V agreement during auditory sentence comprehension. The aim was to explore whether the LAN and P600 effects would vary as a function of the relative perceptual salience associated with *utterance position* and *type of agreement violation* (verb-form). Previous ERP studies investigating the processing of agreement have shown that different aspects of experimental design (e.g., syntactic complexity of the stimuli) can influence the on-line computation of agreement information (Molinaro et al., 2011). However, the possibility that perceptual salience may influence the computation of S-V agreement has not until now been systematically explored. Given the findings from previous S-V agreement studies, we hypothesized that S-V agreement violations will elicit LAN and/or P600 effects. However, we further hypothesized that the effect size of these effects would be moderated by both *utterance position* (medial versus final) and *type of agreement violation* (errors of omission versus commission). More specifically, we predicted that the effects would be more robust for the more perceptually salient conditions (errors of commission and utterance-final position) than for their counterparts.

Results for the overall Grammaticality effect, with all conditions collapsed, showed that S-V agreement violations elicited a bilateral negativity with an anterior-central distribution, in the early 130–210 ms time window, followed by a positivity in the 350–590 ms time window with a centro-posterior distribution. Based on the latency and scalp distribution of the negativity, we interpret the negativity to be an anterior negativity (AN) which has been traditionally taken to reflect similar processes to those reflected by the LAN—i.e., detection of morphosyntactic violations (Friederici et al., 1993; Hagoort et al., 2003; Bornkessel and Schlesewsky, 2006). We also interpreted the positivity to be a P600 effect, which has been traditionally taken to reflect repair, reanalysis or recovery from ungrammatical sentences (Osterhout and Holcomb, 1992; Osterhout and Mobley, 1995; Friederici et al., 1996; Kolk and Chwilla, 2007). The bilateral negativity and the later P600 effect observed for S-V agreement violations is in line with previous studies in the auditory modality (Hahne and Friederici, 2002; Hagoort et al., 2003; Shen et al., 2013).

Having established the overall Grammaticality effects, we extracted the two significant time-windows to perform MANOVAs, exploring whether type of agreement violation and utterance position influenced participants' sensitivity to grammaticality. Contrary to our predictions, we found no interactions involving grammaticality in the early (AN) window. However, for the later (P600) window, we did find a significant three-way interaction between Grammaticality, Position and ROI. This interaction arose because the topography of the Grammaticality effect was different for medial versus final positions. Specifically, while central and parietal ROIs showed comparable P600 effects regardless of position, the P600 at frontal sites was larger for the final position compared to the medial position. According to Rugg and Coles (1995), such quantitative differences in the ERP effects suggest that more neural structures were activated during the processing of the stimuli.

These finding thus provide support for the hypothesis that effects of perceptual salience due to utterance position modulate listeners' sensitivity to S-V agreement violations during on-line speech comprehension. The findings are thus broadly in line with the earlier infant perception study by where infants showed a difference in looking times to the grammatical vs. ungrammatical sentences when the verb and morpheme occurred utterance finally (e.g., *Now he* *cries* vs.^*^Now he *cry*) but not when they occurred utterance medially (*He* *cries* *now* vs.^*^He *cry* *now*, Sundara et al., 2011). Sundara et al.'s results suggest that the effect of position in our ERP paradigm may be more clear-cut in infants and possibly young children than they were in the adults tested here.

However, for type of agreement violations, where the verb-form occurred without −S (errors of omission) and where the verb-form occurred with −S (errors of commission), we found no interactions between Verb-form and Grammaticality in either time window. In other words, participants appeared equally sensitive to omission and commission errors. This is, to our knowledge, the first ERP study to directly compare omission and commission errors in the context of S-V agreement (previous studies have either looked at one error type or have collapsed across both error types). Again, it is worth noting that our participants were all adults listening in their first-language in a pristine auditory environment. It remains to be determined whether omission and commission errors are equally salient for other populations such as children or second-language learners, or indeed, whether L1 adults show differential sensitivity if they have hearing impairment or if the acoustic environment is more challenging.

Although we did not find the predicted interaction between Verb-form and Grammaticality, we did note a main effect of Verb-form for both the AN and the P600. That is, irrespective of Grammaticality, brain responses were different depending on the presence or absence of the −s suffix. This is an important finding from a methodological point of view, demonstrating the need to differentiate between ERP effects that reflect sensitivity to grammatical violation as opposed to those reflecting differences in the acoustic properties of the stimulus. As discussed above, a balanced design (cf. Steinhauer and Drury, 2012) is optimal for investigating the overall effect of S-V agreement, but it does not allow for a more fine-grained analysis that disentangles grammatical and type of agreement violations. Previous studies investigating either omission or commission errors (see Table 1) have taken the opposite approach, keeping the grammatical context constant whilst manipulating the Verb-form. This was also the approach we took in our initial analyses (see **Supplementary Analysis**). However, it fails to disentangle grammatical and acoustic effects on the ERP. Fortunately, the balanced design of our study allowed us to reframe the analysis, contrasting the response to the same verb form in different grammatical contexts, and treating Grammaticality and Verb-form as orthogonal as opposed to confounded factors.

### Implications for the interpretation of the LAN and P600 effects

This study does not allow us to resolve the debate on the processes underlying sentence comprehension. However, it is worth considering how our findings might be incorporated into existing theoretical accounts for the functional interpretation of the LAN/P600 components. As we discussed earlier in the introduction, the functional interpretation of these ERP components has been challenged by reports from agreement processing studies where the realizations of these components has been shown to vary, especially the LAN (for discussion, see Tanner and Van Hell, 2014; Tanner, 2015; Dröge et al., 2016). We argued that these inconsistencies may in part be due to confounding influence the ERP effects during the LAN/AN time window. Importantly, our analysis collapsing across all conditions revealed both AN and P600 effects indicating that listeners detected the morphosyntactic violation and engaged in syntactic re-analysis. The comparisons we conditions represents what Steinhauer and Drury (2012) have referred to as a “balanced” design with the same noun and verb forms occurring equally across conditions such that all confounding factors average out. Notably, the two other studies adopting a balanced design (De Vincenzi et al., 2003; Hasting and Kotz, 2008) also reported a biphasic LAN/P600 response, indicating that the LAN is a robust response to agreement violations if confounding factors are eliminated.

What is interesting, however, is that when we further explored effects of perceptual salience we observed that utterance position effects only influenced S-V agreement processing in the later (P600) time window and not in the earlier (AN) window. This is arguably consistent with the modular-specific model otherwise known as the serial/syntactic-first view (Friederici, 2002). According to this view, syntactic and non-syntactic information interact at a later stage of sentence reanalysis, rather than during the assignment of thematic roles. As a result, P600 effects may vary depending on the non-syntactic information available during sentence re-analysis, whereas the LAN/AN is not expected to vary. The alternative interactive models would predict that effects of perceptual salience should affect both stages of morpho-syntactic processing and syntactic re-analysis given that information about the perceptual salience of the violation is available at every stage of processing (Bornkessel and Schlesewsky, 2006; Pickering and Garrod, 2013). Since we did not observe any significant effects of perceptual salience in the early time window, our data appears to be in support of Friederici's (2002) neurocognitive model of sentence comprehension.

Overall, our results are in line with studies that have reported gradient P600 effects as a result of different agreement-violation manipulations (e.g., Coulson et al., 1998; Nevins et al., 2007). These studies also suggest that the salience of the agreement-violation (e.g., due to type of morphological feature) influences sentence processing. The difference is that, unlike the previous studies, the present study explored the effects of auditory perceptual salience during S-V agreement processing. Our study is therefore the first to show that relative perceptual salience, due to utterance position effects, interacts with syntactic processing during on-line processing of S-V agreement violation and that this interaction happens during the later stage of syntactic re-analysis.

## Conclusions

Studying language-related ERPs in the auditory modality is more ecologically valid for understanding the factors and processes that underlie speech comprehension. However, it raises a number of issues and brings several challenges that are not present with visual presentation. In this study, we explored the possibility that perceptual salience related to *utterance position* (medial vs. final) and *type of agreement violation* (errors of omission vs. commission) influences the computation of S-V agreement violation during speech comprehension. We found significant differences in the ERPs of native-speaking adults for violations occurring in utterance-medial versus utterance-final positions but did not find any significant differences for errors of omission versus errors of commission. We also showed that balanced experimental designs are important, especially in auditory ERP studies where grammaticality effects may be confounded with acoustic differences of the stimuli. The current findings from this study therefore highlight the importance of deconfounding grammaticality effects on ERPs from acoustic and prosodic differences in the stimuli as this has implications for the interpretation of the ERP components associated with morphosyntactic processing. The methodological advances outlined in this paper will be critical in future studies investigating other populations in which perceptual effects might be expected to have more of an impact on agreement processing.

## Author contributions

SD, Designed the experiment, collected and analyzed data, wrote up the paper. CK, Substantial contribution to data analysis, feedback on content, feedback on overall paper for submission. VP, Substantial contribution to experimental design, analysis and feedback on overall paper for submission. JB, Substantial feedback on experimental design, data analysis, and content. KD, Substantial contribution to the design of the experiment, theoretical issues addressed in the study, feedback on the overall paper for submission.

### Conflict of interest statement

The authors declare that the research was conducted in the absence of any commercial or financial relationships that could be construed as a potential conflict of interest.
