# Supplementary material for: Effects of Type of Agreement Violation and Utterance Position on the Auditory Processing of Subject-Verb Agreement: An ERP Study
Source: Front Psychol. 2016 Aug 30;7:1276. doi: 10.3389/fpsyg.2016.01276 (PMC5003887; doi:10.3389/fpsyg.2016.01276)
Supplement: Supplementary file 1 [file DataSheet1.DOCX]

Supplementary Material

Effects of type of agreement violation and utterance position on the auditory processing of subject-verb agreement: An ERP study

**Sithembinkosi Dube*, Carmen Kung, Varghese Peter, Jon Brock, Katherine Demuth**

*** Correspondence:** Sithembinkosi Dube: [sithembinkosi.dube@mq.edu.au](mailto:sithembinkosi.dube@mq.edu.au)

# Supplementary Analysis

This supplementary analysis is based on the target verb manipulations shown in **Supplementary Table 1**.

**Supplementary Table 1:** Experimental design and example of stimuli comparisons for analysis

| **Utterance position** | **Type of agreement violation** | **Example of stimuli** |
| --- | --- | --- |
| ***Medial*** | ***Omission*** | The boy often *cooks/*cook* on the stove |
|  | ***Commission*** | The boys often *cook/*cooks* on the stove |
| ***Final*** | ***Omission*** | The boy often *cooks/*cook* |
|  | ***Commission*** | The boys often *cook/*cooks* |

*Ungrammatical verb forms are marked in asterisks

In these comparisons, grammatical and ungrammatical verbs in utterance-medial and utterance-final position differed as a result of the inflection on the target verb. While these comparisons controlled for the context preceding the target verb, the acoustic properties of the verbs (presence or absence of the –S sound) were confounded with grammaticality (type of agreement violation: errors of omission and errors of commission). We however report the results from the target verb manipulation analysis for comparison, because this is what previous S-V agreement studies using the auditory modality often do (see for example, Shen, Staub and Sanders 2013). The aim of this supplementary analysis, therefore, is to highlight the importance of using balanced experimental designs, which can allow us to analyze ERP responses to speech stimuli without confounding acoustic with grammaticality effects. In the following paragraphs, we present results of the target verb comparisons as revealed by the repeated-measures multivariate analysis of variance (MANOVA).

**Results**

**Effects of type of agreement and utterance position**

After extracting the mean amplitude for the negative cluster (130-210 ms) and the positive cluster (350-590 ms) from the cluster-based permutation test, we explore the effects of perceptual salience using repeated-measures multivariate analysis of variance (MANOVA). Within each time window, we entered Position (medial, final), Grammaticality (grammatical, ungrammatical), Type of agreement violation (singular [i.e. omission], plural [i.e. commission]), and Region of interest (nine regions of interest [ROI]) as within-subject factors. Results of the MANOVA are summarized in **Supplementary Table 2** and the grand averaged ERP waveforms for the grammatical and ungrammatical trials (errors of omission vs. commission) in the utterance-medial and utterance-final position are illustrated in **Supplementary Figures 10** & **Figure 11**.

**Table S2**: **Omnibus MANOVA results across the 130-210 ms, and 350-590 ms time windows**

|  | **130-210ms** | | **350-590ms** | |
| --- | --- | --- | --- | --- |
| Effects | Pillai’s  Trace | F-value | Pillai’s  trace | F-value |
| Type (1,19) | - | - | - | - |
| Pos. (1,19) | - | - | .236 | 5.860* |
| Gram (1,19) | .459 | 16.117*** | .380 | 11.642** |
| Type. * Pos (1,19) | - | - | - | - |
| Type. * Gram (1,19) | .202 | 4.802* | .353 | 10.365** |
| Pos. * Gram (1,19) | - | - | - | - |
| Type.* Pos.*Gram (1,19) | - | - | .234 | 5.807* |
| Type.* ROI (8, 152) | - | - | - | - |
| Pos. *ROI (8, 152) | .686 | 3.280* |  |  |
| Type.*Pos.*ROI (8, 152) | - | - | - | - |
| Gram. * ROI (8, 152) | - | - | - | - |
| Type. * Gram.* ROI (8, 152) | .710 | 3.672* |  |  |
| Pos.*Gram.* ROI. (8, 152) | - | - | .705 | 3.593* |
| Type.*Pos.*Gram.*ROI (8, 152) | - | - | - | - |

Degrees of freedom are reported in parentheses. Pos. = Position, Gram. = Grammaticality, ROI = Regions of interest. ****p* < .001; ***p* < .05; **p* = .05

**
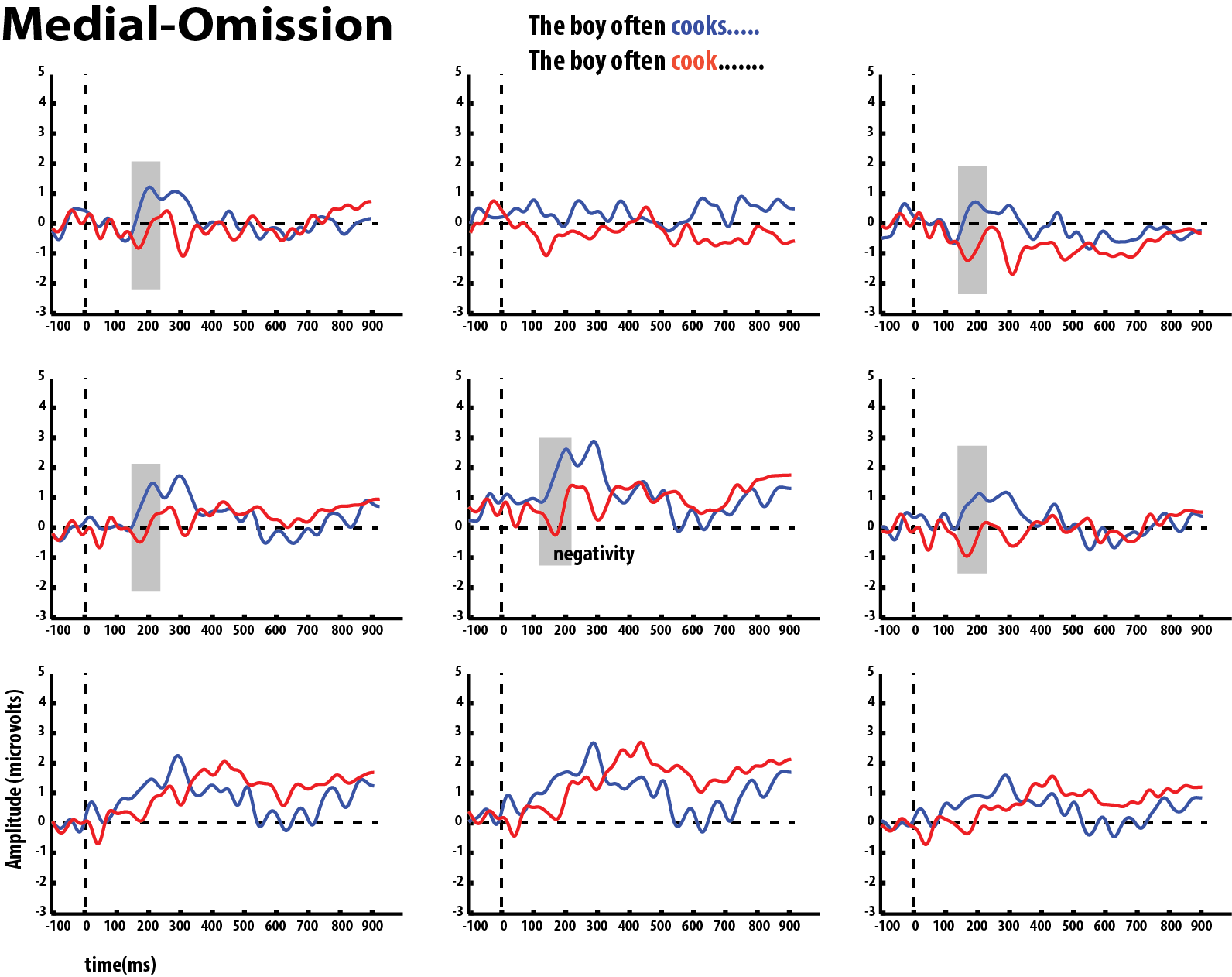
**

**
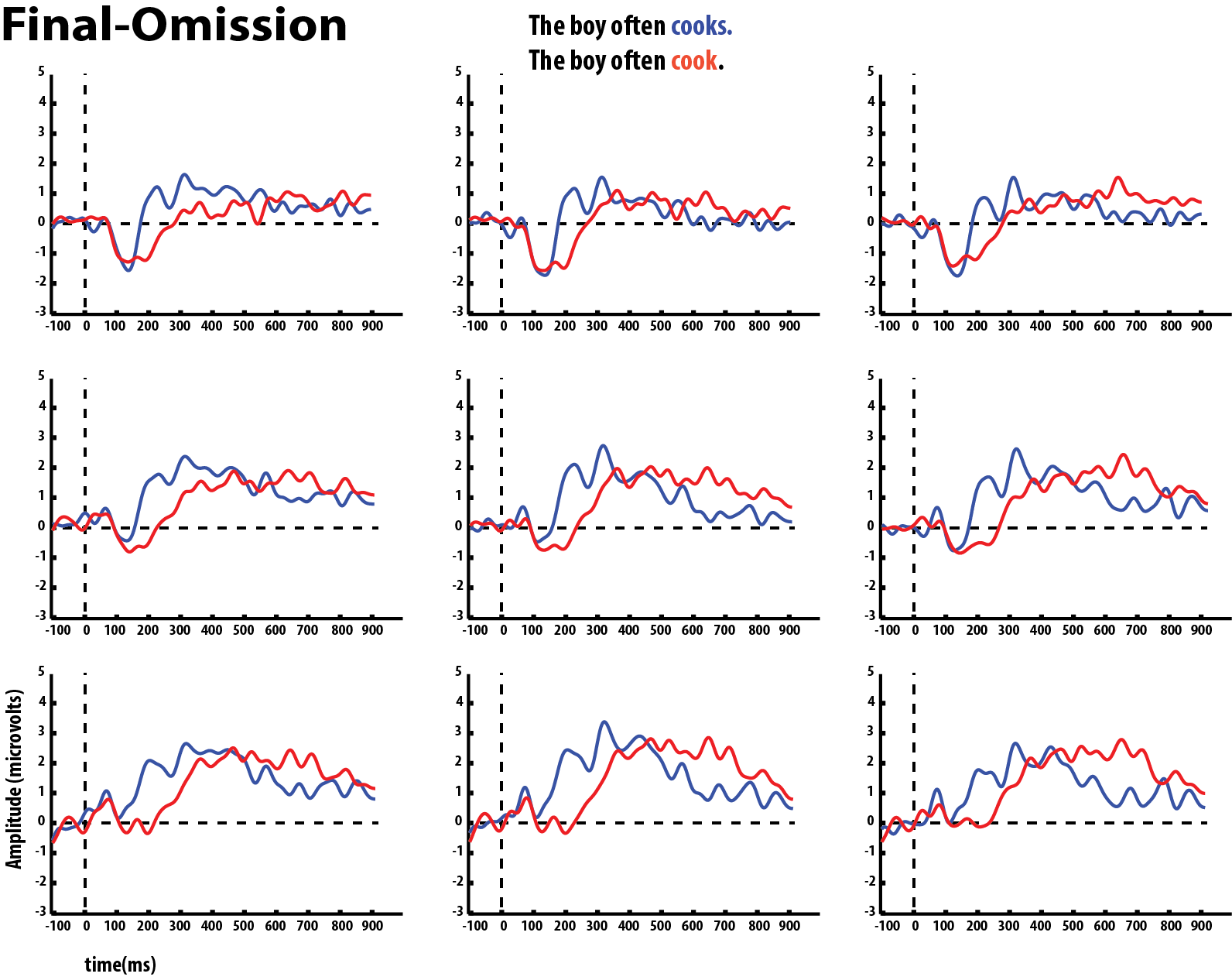
**

**Supplementary Figure 10:** Grand average event-related potentials elicited by errors of omission (red) and correct verb (blue) in medial and final position. Grey bar highlights the significant time-window for the negativity effect.

**
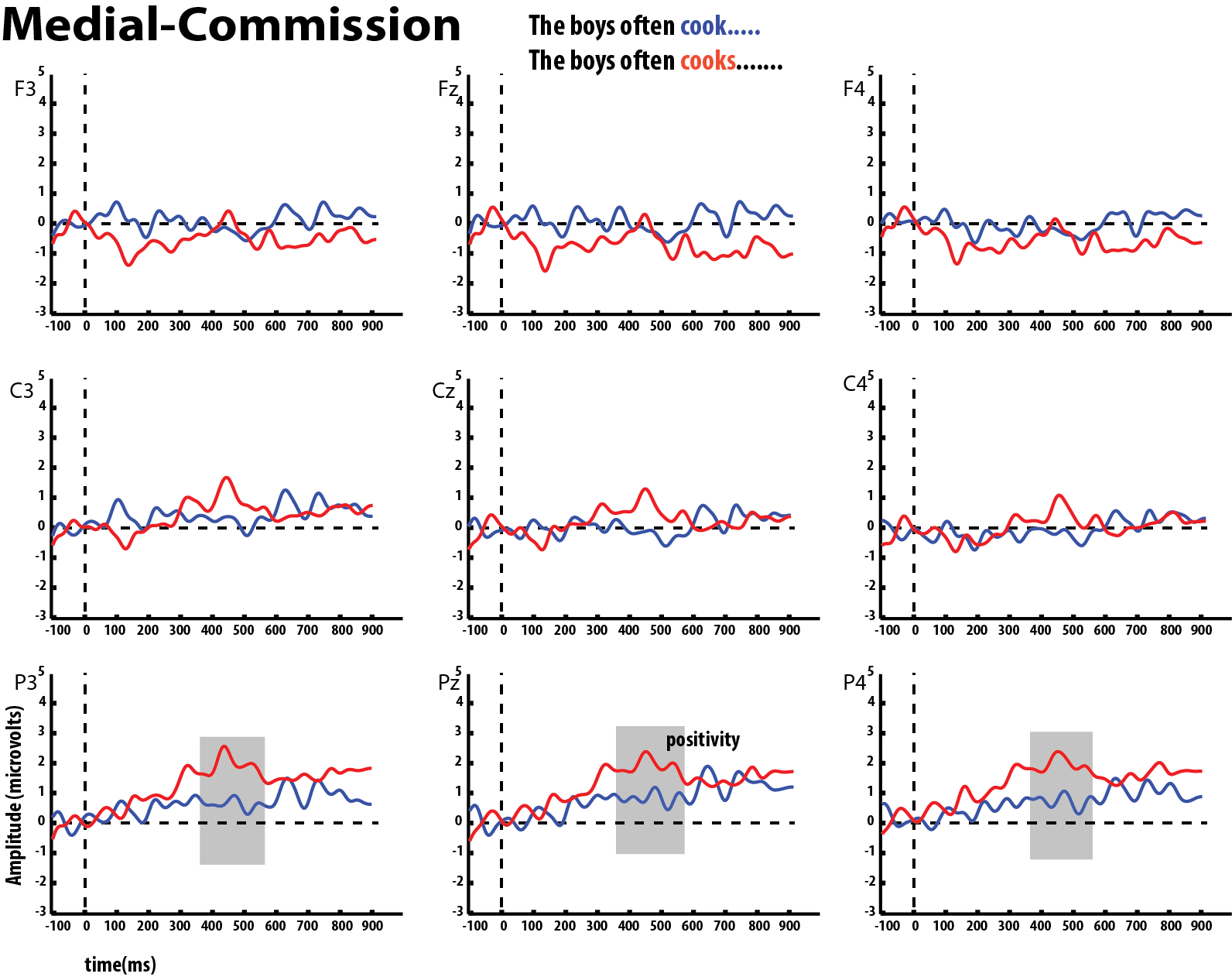
**

**
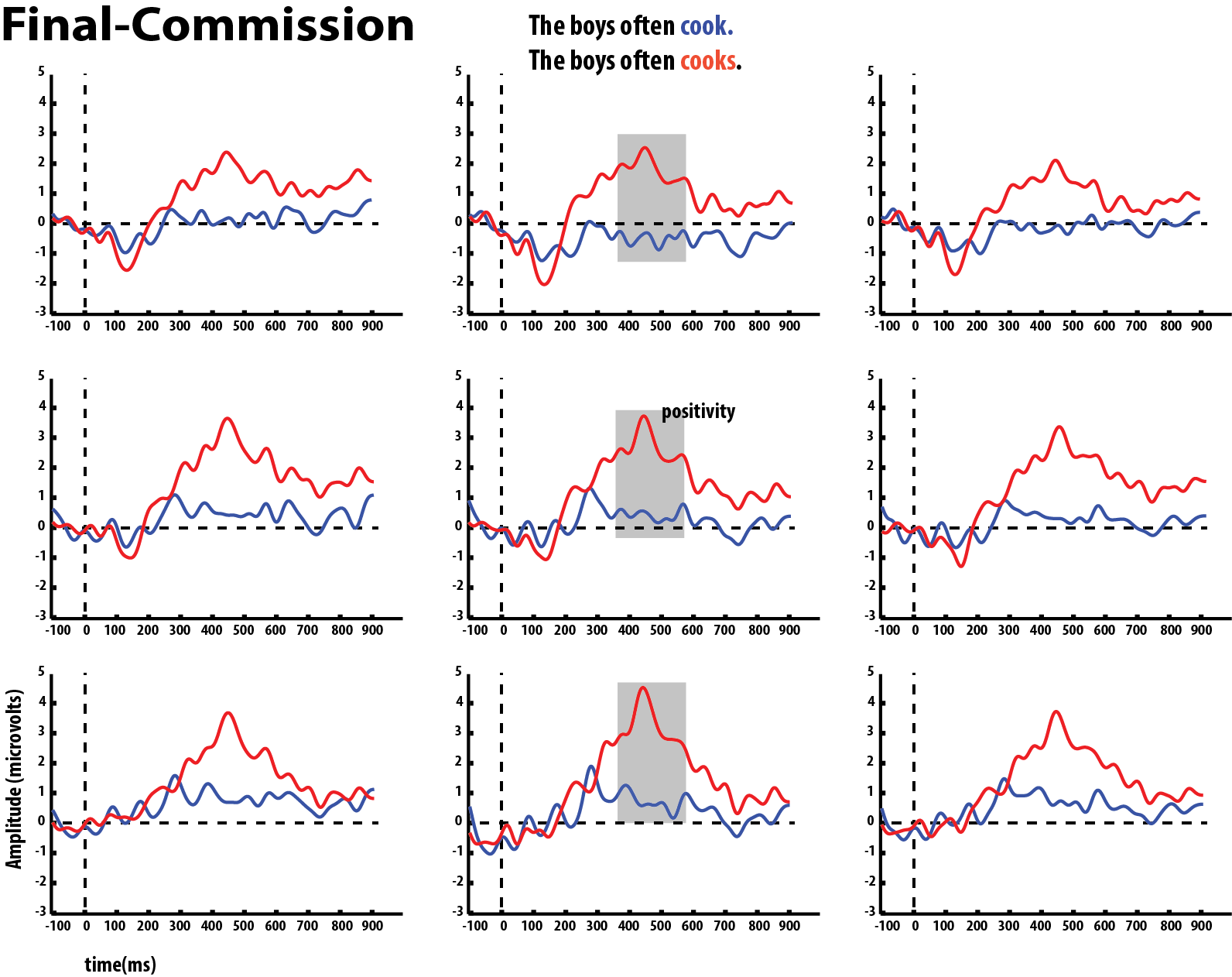
**

**Supplementary Figure 11:** Grand average event-related potentials elicited by errors of commission (red) and correct verb (blue) in medial and final position. Grey bar highlights the significant time-window for the negativity effect.

**ERP results in the 130-210 ms time window**

The statistical analysis for this time window showed a main effect of Grammaticality and interactions between Type of agreement and Grammaticality, between Position and ROI, and between Type of agreement violation, Grammaticality, and ROI (Pillai’s trace and F values are given in Table S2). The interaction between Type and Grammaticality suggests that, in this time-window, the effect of Grammaticality differed depending on the Type of agreement violation. Follow up pairwise t-tests revealed that the mean amplitude of the negativity for ungrammatical conditions was significantly greater for errors of omission (M = -0.314 µV, SE = 0.177) than errors of commission (M = -0.007 µV, SE = 0.188), t (19) = -2.543, p < .005. This shows that this interaction was driven by the negativity elicited by errors of omission

In addition to the two-way interaction, the three-way interaction between Type, Grammaticality, and ROI suggests that the mean amplitudes of the electrodes differed depending on Grammaticality and Type. To test this, follow-up MANOVAS were performed on each ROI with Type and Grammaticality as within-subject factors. Results indicated that the interaction was significant in the central-mid region (Pillai’s trace = .205, F(1,19) = 4.904, p < .05), posterior-left region, (Pillai’s trace = .255, F(1,19) = 6.505, p < .05) posterior-mid region, (Pillai’s trace = .301, F(1,19) = 8.185, p < .05) and the central right region (Pillai’s trace = .279, F(1,19) = 7.346, p < .05). Further pairwise comparisons showed that the mean-amplitude differences for the ungrammatical conditions were only significant for errors of omission in the central-mid region (M = 1.259 µV, SE = 0.400), t (19) = 3.143, p < .005; and posterior-mid region (M = 0.664 µV, SE = 0.292), t (19) = 2.274, p < .05.

Finally, the interaction between Position and ROI suggests that the mean amplitude of the electrodes differed depending on Position. Follow up pairwise t-tests revealed that the mean amplitude of the negativity in the central-mid region was significantly greater for medial (M = -.463 µV, SE = .274) than final positions (M = -.095 µV, SE = .230), t (19) = -2.543, p < .05. This shows that the negativity observed in the cluster-based permutation test was driven by type of violation (errors of omission) in the medial position. This is also reflected in the grand averaged ERP waveforms for the grammatical and ungrammatical trials (errors of omission vs. commission) in the utterance-medial and utterance-final position in **Figure 10** and **Figure 11**. In overall, the interactions observed in this time window indicate a negativity for errors of omission in the medial condition.

**ERP results in the 350-590 ms time window**

The statistical analysis for this time window showed a main effects of Grammaticality and Position as well as interactions between Type and Grammaticality, and between Type, Position and Grammaticality, and between Position, Grammaticality, and ROI (Pillai’s trace and F values are given in Table S2). The interaction between Type and Grammaticality suggests that, in this time-window, the effect of Grammaticality differed depending on the Type of agreement violation. Follow up pairwise t-tests revealed that the mean amplitude of the positivity for ungrammatical conditions was significantly greater for errors of commission (M = 1.372 µV, SE = 0.228) than errors of omission (M = 0.860 µV, SE = 0.244), t (19) = 3.152, p < .005. This shows that this interaction was driven by the positivity elicited by errors of commission.

In addition, the three-way interaction between Type, Position, and Grammaticality suggests that the effect of the Grammaticality also differed depending on Type of agreement violation and Position. To test this, follow-up MANOVAS were performed on grammatical and ungrammatical conditions with Type, Position and ROI as within-subject factors. The analysis showed main effects of Position and Type and a further three way interaction between Type, Position and ROI (Pillai’s trace = .719, F(1,19) = 3.847, p < .05) in the ungrammatical condition. Further analysis performed on each ROI with Type and Positon as within factors revealed that the interaction was significant in the front-left region (Pillai’s trace = .290, F(1,19) = 7.755, p < .05). Follow up pairwise t-tests revealed that the mean amplitude of the positivity for ungrammatical conditions was significantly greater for errors of commission in the utterance-final position (M = 1.520 µV, SE = 0.447) than in the utterance-medial position (M =0.684 µV, SE =0 .286), t (19) = -3.399, p < .005. This shows that in the 350-590 ms time window, errors of commission in the utterance-final position elicited a robust positivity. This indicates that the positivity observed in the overall cluster-based permutation was driven by the errors of commission in the utterance final position.

Furthermore, the other three-way interaction between Position, Grammaticality, and ROI suggests that the mean amplitude of the positivity also differed depending on Position and Grammaticality. To test this, follow-up MANOVAS were performed on each ROI with Position and Grammaticality as within-subject factors. Results indicated that the interaction was significant in the front-mid region (Pillai’s trace = .296, F(1,19) = 7.982, p < .05), front-left region (Pillai’s trace = .224, F(1,19) = 5.343, p < .05), central-mid region, (Pillai’s trace = .190, F(1,19) = 4.459, p < .05) and posterior-mid region, (Pillai’s trace = .279, F(1,19) = 7.236, p < .05). Further pairwise comparisons showed that the medial and final ungrammatical conditions were only significantly different for the errors of commission in the front-mid region (M = 2.079 µV, SE = .657), t (19) = 3.152, p < .005, central-mid region, (M = 1.218 µV, SE = 0.529), t (19) = 3. 038, p < .05, posterior-mid region, (M = 1.027 µV, SE = .404) (t (19) = 2.657, p < .05, and the front-left region (M = 1.520 µV, SE = 0.444) (t (19) = 3.399, p < .005. We interpreted these results to show that ungrammatical conditions in the final position elicited a broadly distributed positivity. This pattern is reflected in the grand averaged ERP waveforms for grammatical and ungrammatical trials (errors of omission vs. commission) in the utterance-medial and utterance-final position in Figure 10 and Figure 11.

Overall, we interpreted the interactions observed in this time window to indicate that the amplitude and distribution of the positivity was influenced by the additive perceptual salience due to overtness of the violation and utterance-final lengthening. Thus, these result supported our hypothesis that the more perceptually salient conditions would yield more robust ERP effects than the less perceptually salient conditions. In particular, errors of commission in the utterance-final position evoked a larger P600 effect and broader scalp distribution compared with those at the utterance-medial position, and compared with the errors of omission at the utterance-final position. Furthermore, that errors of omission in the utterance-medial position elicited only a bilateral anterior negativity while the errors of commission at the same position elicits only a P600 effect with a centro-posterior distribution. We concluded that these results indicated that listeners were more sensitive to ungrammatical sentences with errors of commission than errors of omission due to greater perceptual salience of the overt violation and that this sensitivity was greater when the S-V agreement errors occurred utterance-finally than utterance-medially.

However, as argued in the main paper, the comparisons used for this analysis confounded grammaticality with differences in the acoustic content following the verb stem, in terms of both the presence/absence of the –s and the timing of the subsequent word. Thus it is possible that “grammaticality” effects on ERPs may arise even when participants are insensitive to the grammatical violation. This could have been the case with the negativity effects observed in the medial conditions, given that these effects were no longer significant in the alternative analysis where we manipulated the context whilst keeping the verb inflection constant across the singular and plural conditions. (e.g., *The boy often cooks on the stove* vs. *The boys often *cooks on the stove*). Although it could be argued that the context manipulation could itself present as an acoustic confound affecting the pre-stimulus baseline, this was minimized by the intervening adverb (see Steinhauer and Drury (2012) for discussion on effects of context/target manipulation on syntactic violation processing).

We therefore recommend that future ERP studies using inflectional S-V agreement should adopt similar balanced designs as used in our study. By doing so, we are able to deconfound grammaticality effects on ERPs from acoustic differences in the stimuli. This is important as it has implications for the interpretation of the ERP components associated with morphosyntactic processing.
